# Stereocilia-staircase spacing is influenced by myosin III motors and their cargos espin-1 and espin-like

**DOI:** 10.1038/ncomms10833

**Published:** 2016-03-01

**Authors:** Seham Ebrahim, Matthew R. Avenarius, M’hamed Grati, Jocelyn F. Krey, Alanna M. Windsor, Aurea D. Sousa, Angela Ballesteros, Runjia Cui, Bryan A. Millis, Felipe T. Salles, Michelle A. Baird, Michael W. Davidson, Sherri M. Jones, Dongseok Choi, Lijin Dong, Manmeet H. Raval, Christopher M. Yengo, Peter G. Barr-Gillespie, Bechara Kachar

**Affiliations:** 1 Laboratory of Cell Structure and Dynamics, National Institute on Deafness and Other Communication Disorders, National Institutes of Health, Bethesda, Maryland 20892, USA; 2 Oregon Hearing Research Center and Vollum Institute, Oregon Health & Science University, Portland, Oregon 97239, USA; 3 National High Magnetic Field Laboratory and Department of Biological Science, Florida State University, Tallahassee, Florida 32310, USA; 4 Department of Special Education and Communication Disorders, University of Nebraska-Lincoln, Lincoln, Nebraska 68583, USA; 5 Department of Public Health and Preventive Medicine, Oregon Health and Science University, Portland, Oregon 97239, USA; 6 Genetic Engineering Core, National Eye Institute, National Institutes of Health, Bethesda, Maryland 20892, USA; 7 Department of Cellular and Molecular Physiology, Penn State University College of Medicine, Hershey, Pennsylvania 17033, USA

## Abstract

Hair cells tightly control the dimensions of their stereocilia, which are actin-rich protrusions with graded heights that mediate mechanotransduction in the inner ear. Two members of the myosin-III family, MYO3A and MYO3B, are thought to regulate stereocilia length by transporting cargos that control actin polymerization at stereocilia tips. We show that eliminating espin-1 (ESPN-1), an isoform of ESPN and a myosin-III cargo, dramatically alters the slope of the stereocilia staircase in a subset of hair cells. Furthermore, we show that espin-like (ESPNL), primarily present in developing stereocilia, is also a myosin-III cargo and is essential for normal hearing. ESPN-1 and ESPNL each bind MYO3A and MYO3B, but differentially influence how the two motors function. Consequently, functional properties of different motor-cargo combinations differentially affect molecular transport and the length of actin protrusions. This mechanism is used by hair cells to establish the required range of stereocilia lengths within a single cell.

## Abstract (web summary)

Stereocilia of the inner ear have graded heights that are thought to be regulated by the myosin-III family members MYO3A and MYO3B. Here the authors identify espin-1 and espin-like (ESPNL) as cargo that differentially influence the functions of both motors to regulate stereocilia length.

## Graphical abstract

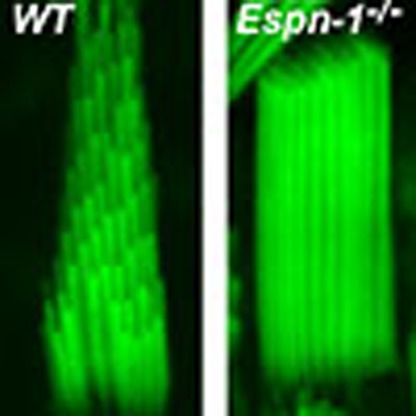

Cellular actin-rich protrusions enable many biological functions, including migration, exploration and response to external stimuli^1^,^2^. Such functional diversity is made possible by variations in the dimensions, dynamics and positioning of actin structures within cells. In the inner ear, sensory hair cells are initially decorated with microvilli; these are remodelled during development to generate rows of stereocilia, which have precisely graded heights and form the mechanically sensitive hair bundle^3^. The bundle’s staircase organization is vital for its role in converting mechanical stimuli like sound to neural signals. Remarkably, stereocilia within a bundle can range from less than 1 to over 100 μm long^4^. The formation and maintenance of such an extraordinary range of lengths must require localized regulatory mechanisms within each stereocilium, and modulation must occur differentially across adjacent rows^5^,^6^,^7^. Although mechanisms of stereocilia height regulation are largely unknown, the significance of the problem is highlighted by the large number of deaf mouse mutants with abnormal stereocilia morphology^8^.

The dimensions of the parallel actin filament bundles that make up the stereocilia core are regulated by actin-binding proteins^9^,^10^, as well as unconventional myosin motors and their cargos^11^,^12^,^13^. Complexes of either of two unconventional myosins, MYO3A and MYO3B, together with their actin-regulating cargo ESPN-1, are candidates for controlling stereocilia lengths, based on several lines of evidence. Each of the three proteins localizes to the distal tips of stereocilia, the sites of actin polymerization, in a length-dependent distribution^13^,^14^,^15^,^16^,^17^,^18^. Second, ESPN-1 is transported by MYO3A or MYO3B to tips of filopodia in cultured cells, as well as to stereocilia tips, where motor and cargo synergistically elongate these actin structures^13^,^14^. Third, mutations in *MYO3A* have been linked to DFNB30, a late-onset, progressive hearing loss^19^, and mutations in the *ESPN* gene, which encodes ESPN-1 as well as shorter splice forms, are associated with hearing and vestibular abnormalities^20^,^21^. In this study, we investigated the roles of myosin-III paralogs and ESPN-1 in stereocilia formation using *Myo3a*^^−/−^^, *Myo3b*^−/−^ and *Espn-1*^−/−^ mice. In addition, we identified a novel myosin-III cargo, espin-like (ESPNL), which appears early in bundle development and is essential for normal auditory function. We show that these two myosins and their two cargos form a system that influences the number and the length of actin protrusions, and is critical for the formation of the normal stereocilia-staircase structure.

## Results

### Loss of staircase organization in *Espin-1* null hair bundles

**Figure 1:**
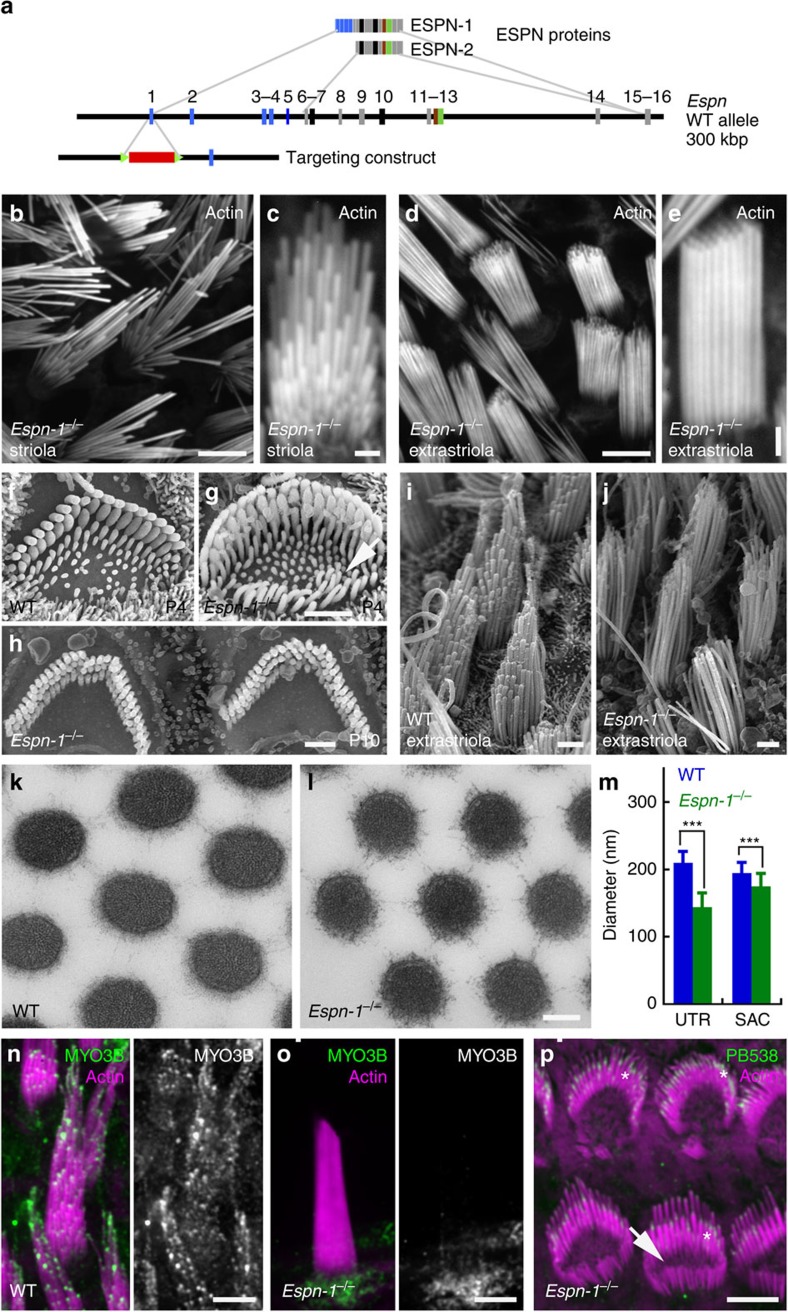
Altered length and width regulation in *Espn-1*^−/−^ mice. (**a**) Targeting strategy. PGK-Neo cassette targeted exon 1, which includes the translation start for ESPN-1; translation start for short ESPN isoforms is in exon 6. (**b**,**c**) Phalloidin-labelled stereocilia in the striola of *Espn-1*^−/−^ utricles, imaged with confocal fluorescence microscopy, appear normal. (**d**,**e**) Phalloidin-labelled stereocilia in an extrastriolar region of *Espn-1*^−/−^ utricles are of nearly uniform lengths. (**f**) SEM image of WT cochlear outer cell hair bundles. (**g**) SEM of P4 *Espn-1*^−/−^ outer hair cells shows elongated protrusions opposite the bundle (arrow). (**h**) Extraneous protrusions disappear from *Espn-1*^−/−^ outer hair cells by P10. (**i**) SEM image of WT utricle extrastriolar bundles. (**j**) SEM of extrastriolar bundles of *Espn-1*^−/−^ utricle; stereocilia are of nearly uniform length. (**k**,**l**) Transmission electron micrographs show that extrastriolar stereocilia of *Espn-1*^−/−^ utricle (**l**) have a smaller diameter than those of WT utricles (**k**). (**m**) Diameter of utricle (UTR) and saccule (SAC) stereocilia. Mean±s.d.; *n*=43 (WT utricle), 163 (*Espn-1*^−/−^ utricle), 134 (WT saccule) and 168 (*Espn-1*^−/−^ saccule). Differences are significant at *P*<0.001 (Student's *t*-test). (**n**) MYO3B targets to stereocilia tips of utricle bundles of WT mice. (**o**) MYO3B no longer targets to tips of *Espn-1*^−/−^ stereocilia. (**p**) PB538 anti-ESPN-1 antibody detects antigen at some stereocilia tips (asterisks). Elongated protrusions (arrow) are visible in inner hair cells. Scale bars (**b**,**d**,**m**–**o**), 5 μm; (**c**,**e**–**j**), 2 μm; and (**k**,**l**), 100 nm.

To test whether transport of ESPN-1 by myosin-III affects stereocilia length, we used conventional gene targeting at the *Espn* locus to generate a mouse line (*Espn-1*^−/−^) that lacks *Espn-1*, but expresses short *Espn* isoforms (Fig. 1a). Stereocilia in the organ of Corti of *Espn-1*^−/−^ mice appeared normal, with the exception of transient elongated microvillar-like protrusions on the hair-cell surface, opposite to the hair bundle (Fig. 1g); these protrusions disappeared by P10 (Fig. 1h). *Espn-1*^−/−^ mice had normal hearing (Supplementary Fig. 1).

By contrast, ablation of *Espn-1* produced stereocilia length abnormalities in specific regions of the otolith organs, the utricle and saccule, of vestibular sensory epithelia. These organs are used for detection of linear acceleration in rodents and can be subdivided into striolar and extrastriolar regions (Supplementary Fig. 2). The striola, a region of reduced hair-cell density^22^, is specialized for detection of dynamic (phasic) stimuli; the extrastriolar regions, which include medial and lateral extrastriola, encode tonic stimuli^23^. While hair bundles in the striolar region of the utricle and the saccule had normal morphology in *Espn-1*^−/−^ mice (Fig. 1b,c), extrastriolar hair cells had bundles with a striking reduction in the slope of their staircase (Fig. 1d,e,j), caused both by a shortening of the tallest row of stereocilia in the bundle and elongation of shorter rows. Stereocilia lengths measured from scanning electron microscopy (SEM) images showed that the length difference between tallest and shortest stereocilia in extrastriolar bundles decreased from 10.8±0.9 μm (mean±s.d.; *n*=6 cells) in heterozygotes to 1.9±0.6 μm (*n*=4) in *Espn-1*^−/−^ mice, a reduction of >80%. Moreover, the diameter of vestibular stereocilia in the *Espn-1*^−/−^ mouse was significantly less than those of wild-type (WT) mice (Fig. 1k–m). Nevertheless, *Espn-1*^−/−^ mice showed no overt vestibular abnormalities (Supplementary Fig. 1a–d) and MYO15A was targeted normally (Supplementary Fig. 1f,g).

Because MYO3B lacks the actin-binding tail homology domain 2 (THD2), it cannot travel to distal tips of actin protrusions without interaction with ESPN-1 (ref. 14). Indeed, in *Espn-1*^−/−^ mice, MYO3B was no longer detectable at stereocilia tips of extrastriolar hair cells (Fig. 1n,o). Thus, at least in extrastriolar hair cells, ESPN-1 is required for targeting of MYO3B to stereocilia tips, as well as regulation of stereocilia diameter and staircase formation.

### ESPN and ESPNL are prominent in mouse utricle hair bundles

**Figure 2:**
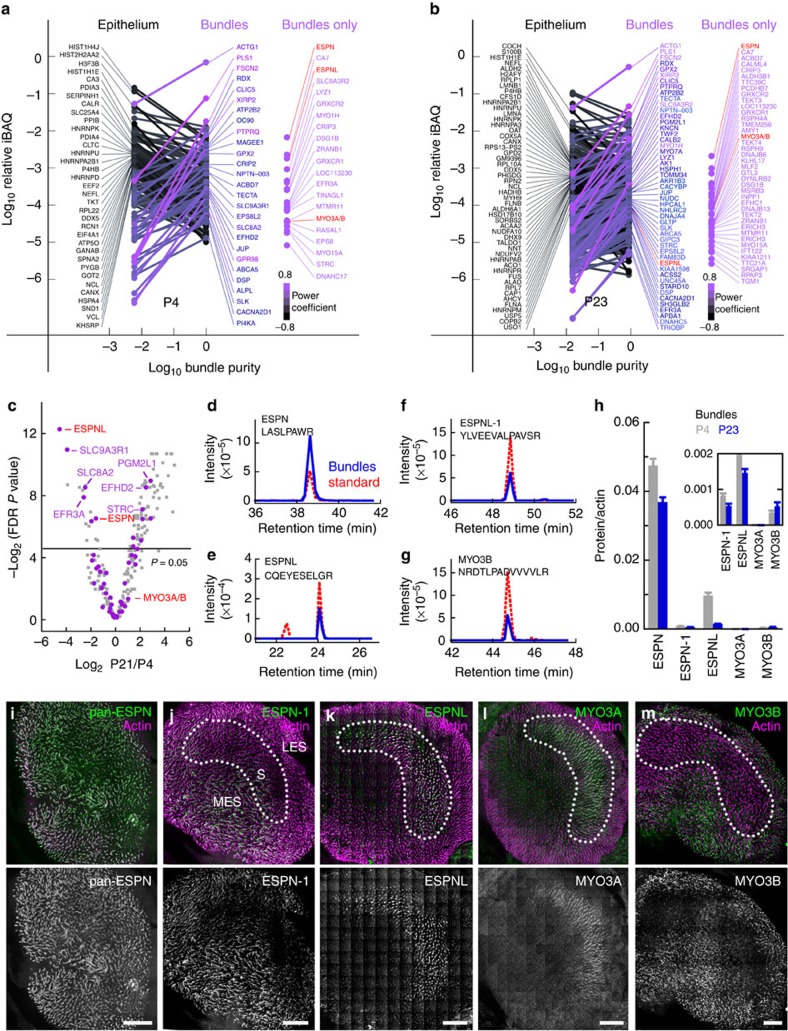
Proteomics of developing mouse hair bundles. (**a**) Mole fractions of proteins in utricular epithelium (left) and hair bundles (right) of P5 mice. Proteins most highly enriched in epithelium are indicated at left and those highly enriched in bundles at right. Hue represents enrichment for each protein. Far right, proteins detected only in bundles. (**b**) Mole fractions of proteins in epithelium (left) and bundles (right) of P23 mice. (**a**,**b**) Derived from four biological replicates of 100 ear-equivalents of hair bundles and 10 whole utricles. (**c**) Volcano plot showing relationship between P23/P5 enrichment (*x* axis) and FDR-adjusted *P* value (*y* axis). Proteins that are enriched fivefold or greater between bundles and epithelium are labelled with purple. (**d**) Targeted MS2 signal for ESPN peptide LASLPAWR (*m/z*=457.2663, 2+ charge state) detected from P23 bundles; 10 fmol of the heavy-isotope-labelled peptide (462.2705, 2+) was included as a standard. (**e**) Targeted MS2 signal for ESPN-1 peptide YLVEEVALPAVSR (723.4036, 2+) detected from P23 bundles; 1 fmol of standard (728.4077, 2+). (**f**) Targeted MS2 signal for ESPNL peptide CQEYESELGR (635.7721, 2+) detected from P23 bundles; 1 fmol of standard (640.7762, 2+). (**g**) Targeted MS2 signal for MYO3B peptide NRDTLPADVVVVLR (522.9703, 3+) detected from P23 bundles; 1 fmol of standard (526.3063, 3+). (**h**) Summarized PRM signal for each protein, normalized to actin; two to three peptides were used for each protein, and four biological replicates were measured for each age (reported as mean±s.d.). While MYO3A was detectable in one sample at P5, its signal was below the assay limit of detection at P23. (**i**–**m**) Low-power images of mouse utricle stained with phalloidin (top, magenta) for actin and specific antibodies (top, green; below, grey). Approximate location of the striola, estimated from examination of the phalloidin channel, is indicated. ESPN-1 (**j**) and MYO3B (**m**) are enriched in extrastriolar regions; ESPNL (**k**) and MYO3A (**l**) are enriched in the striola. LES, lateral extrastriola; MES, medial extrastriola; S, striola. Scale bars, 100 μm. FDR, false discovery rate.

Surprisingly, PB538, an antibody directed against the ankyrin-repeat domain (ARD) of ESPN-1 (ref. 13), exhibited irregular immunoreactivity towards stereocilia tips in *Espn-1*^−/−^ hair bundles (Fig. 1p). To search for ESPN paralogs expressed in hair bundles, we used the twist-off method^10^,^24^ to isolate bundles from developing (∼P4) and young adult (∼P23) mouse utricles^25^. We detected proteins with shotgun mass spectrometry and liquid chromatography-tandem mass spectrometry (MS/MS) using an Orbitrap mass spectrometer, calculating each protein’s approximate molar abundance using relative iBAQ values^10^,^25^. Figure 2a,b shows the abundance of proteins detected in both bundles and epithelium at the two time points; the slope of the connecting lines indicates the bundle-to-epithelium enrichment^25^.

The ESPN-1 paralog ESPNL was highly enriched in hair bundles; on a volcano plot, ESPNL was the most statistically significant P4-enriched protein in the entire data set (Fig. 2c), emphasizing its high concentration in young utricle bundles. The summed splice forms of ESPN were also highly enriched and together were more abundant than ESPNL (Fig. 2a,b). MYO3A and MYO3B were present at low levels, although total myosin-III went up approximately threefold from P4 to P23. Examining peptide intensity that distinguished the two myosin paralogs, we estimated that the fraction represented by MYO3A went from ∼20% at P4 to below the limit of detection in this assay at P23.

For greater quantification accuracy, we used parallel reaction monitoring (PRM), a targeted proteomics modality^26^, to specifically assay ESPN, ESPN-1 and ESPNL in utricle hair-bundle extracts using 2–3 peptides each (Fig. 2d–h). Accurate quantification was assured by using quantified, heavy-isotope-labelled peptides as standards for each assay; assays were also validated by matching MS2 spectra collected at peaks to the appropriate protein in the mouse Ensembl database. By targeted proteomics, ESPN-1 accounted for <2% of the total ESPN; by contrast, ESPNL was 10-fold more abundant at P4 than ESPN-1. While ESPN-1 and total ESPN declined in utricle bundles by less than twofold between P4 and P23, ESPNL decreased sevenfold (Fig. 2h). MYO3B was roughly equal in concentration to ESPN-1 at P4 and P23, while MYO3A was considerably less abundant (only detected in one out of four biological replicates at P4, and none at P23).

Quantification of these proteins in the whole utricle ignores regional variations in expression. Using immunofluorescence in utricular wholemounts, we found that the myosin-III paralogs and their espin-related cargos showed striking enrichment in either striola (ESPNL and MYO3A) or extrastriola (ESPN-1 and MYO3B) zones (Fig. 2i–m). Higher magnification images revealed the boundaries clearly (Supplementary Figs 3–7).

### Localization of ESPNL in hair bundles

**Figure 3:**
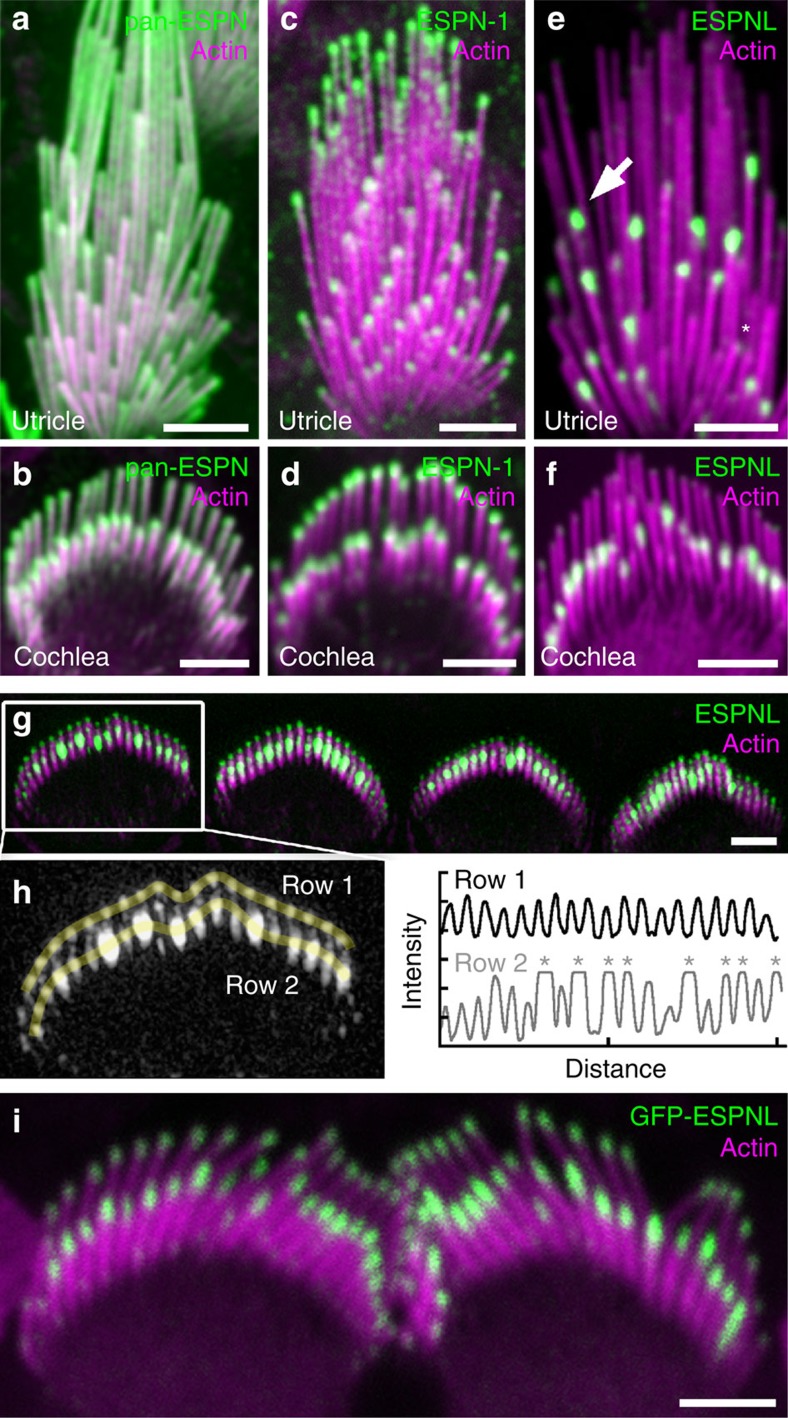
Immunolocalization of ESPN-1 and ESPNL. (**a**,**b**) Pan-ESPN antibody (green) and actin (magenta) labels utricle (**a**) and cochlear outer hair cell (**b**) stereocilia throughout. (**c**,**d**) ESPN-1 antibody labels the tips of utricular (**c**) and cochlear (**d**) stereocilia. (**e**) ESPNL antibody (ab170747) labels some stereocilia tips strongly (arrow), some weakly (asterisk), but does not label tallest stereocilia of utricle hair cell. (**f**) ESPNL antibody labels most but not all stereocilia tips of row 2 in bundle of outer hair cell. (**g**) Structured illumination microscopic image of ESPNL labelling (BG35961) of inner hair-cell bundles. (**h**) Quantification of ESPNL labelling of one hair bundle from (G). (left) Yellow lines indicate quantification trajectories; and (right) uniform labelling in row 1 but saturation of some tip signals in row 2 (asterisks). (**i**) GFP-ESPNL labelling of P0.5 inner hair cells following *in utero* electroporation at E12. Scale bars, 2 μm.

As previously seen^13^,^21^, a pan-ESPN antibody labelled the entire length of auditory and vestibular stereocilia (Fig. 3a,b). The PB538 ESPN-1 antibody labelled stereocilia tips of WT utricles; the signal was more concentrated at tips of taller stereocilia than shorter ones^13^ (Fig. 3c,d). Although this antibody cross-reacts with ESPNL, its reactivity is stronger towards ESPN-1 (Supplementary Fig. 1i–k). An antibody specific to the C-terminus of ESPNL (ab170747; Supplementary Fig. 1j) labelled stereocilia tips in both vestibular and auditory organs (Fig. 3e,f); ESPNL was most concentrated at the tips of second and shorter rows. *In utero* electroporation of GFP-ESPNL (Fig. 3i) and biolistic transfection of mEmerald-ESPNL (Supplementary Fig. 8h,i) confirmed targeting of ESPNL to stereocilia tips, particularly those of the second and shorter rows. Interestingly, in both cochlea and utricle, ESPNL levels were remarkably non-uniform between stereocilia, including adjacent ones of the same length (Fig. 3e–i and Supplementary Fig. 8h,i). In early postnatal cochlea, this variability was most prominent in row 2; structured illumination microscopy (SIM) indicated that row 1 had much smaller but nearly uniform levels of ESPNL (Fig. 3g,h). Here ESPNL was detected with BG35961, also directed against the C-terminus (Supplementary Fig. 9d). In the utricle, labelling of variable intensity was seen in short- and intermediate-length stereocilia (Fig. 3e).

In the utricle, at P0.5, ESPNL was present in all hair bundles, although levels were higher in the striola; ESPNL was nearly gone from extrastriolar bundles by P7.5 and was not detected there at P20.5, despite robust expression in striolar bundles (Supplementary Fig. 8e–g). This result was consistent with the mass spectrometry quantification. In the cochlea, expression of ESPNL was transient, with expression levels falling between P3 and P10 (Supplementary Fig. 8a–d). The ab170747 and BG35961 antibodies reported similar ESPNL distribution in cochlea and utricle.

### ESPNL binds but does not crosslink actin

**Figure 4:**
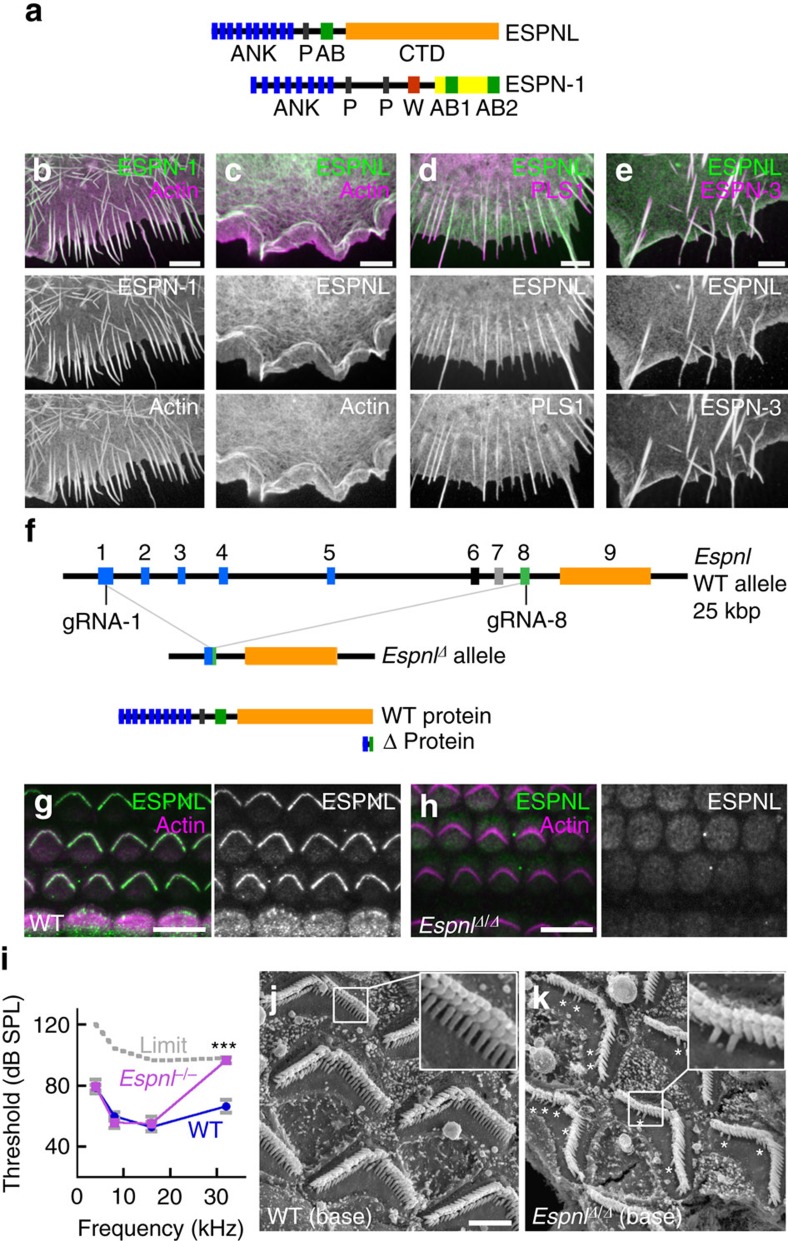
ESPNL functional characterization. (**a**) Domain structure of ESPNL and ESPN-1. Blue, ankyrin repeats (ANK); black, proline-rich domain (P); red, WH2 domain (W); green, common actin-binding region (C); yellow, ESPN actin-binding domain (AB); and orange, ESPNL C-terminal domain (CTD). (**b**) COS7 cells transfected with mCherry-ESPN1 (green), also labelled with phalloidin (magenta). Individual channels shown below. ESPN-1 forms filopodia. (**c**) mCherry-ESPNL localizes with actin structures but does not induce filopodia. (**d**) ESPNL localizes to filopodia induced by the actin-bundling protein GFP-PLS1. (**e**) ESPNL localizes to filopodia induced by GFP-ESPN-3. (**f**) CRISPR targeting strategy. gRNA-1 targeted exon 1 and gRNA-2 targeted exon 8; one of 16 alleles was a deletion between those two exons, eliminating ∼20 kbp of genomic DNA. The Δ protein is hypothetical; we do not have evidence that this truncated product is even made. (**g**,**h**) BG35961 anti-ESPNL antibody labels WT cochlear hair bundles (**g**) but not those of *Espnl^Δ/Δ^* mice (**h**). (**i**) ABRs showing high-frequency hearing loss in 30-day mice. Mice with *Espnl^Δ/Δ^*, *EspnlΔ/A* and *Espnl^A/A^* genotypes had similar ABRs and were grouped together as *Espnl−/−* (*n*=13; WT, *n*=19; displayed as mean±s.d.). ***P<10^−5^ (Student’s *t*-test). (**j**) SEM of basal cochlear outer hair cells from WT mice. Note three rows of stereocilia in each bundle. (**k**) SEM of basal cochlear outer hair cells from *EspnlΔ/Δ* mice. Bundles only have two rows of stereocilia except for scattered row three stereocilia (asterisks). Scale bars, (**b**–**e**,**j**) 2 μm; and (**g**,**h**) 10 μm.

While ESPN-1 and ESPNL are only 26% identical, ESPNL contains a 27-residue sequence in the middle of the protein that shares 74% sequence homology with a region within the actin-binding module of ESPN-1 (Fig. 4a). Indeed, when expressed in COS7 cells, tagged ESPNL co-localized strongly with the actin filament network (Fig. 4c) but unlike ESPN-1 (Fig. 4b), did not induce the formation of actin bundles or protrusions. When co-expressed with known actin-crosslinking proteins PLS1 (ref. 27) and ESPN-3 (ref. 17), ESPNL targeted to bundled actin (Fig. 4d,e). ESPNL thus binds to but does not crosslink actin, presumably because it lacks the additional actin-binding modules present in ESPN-1.

### High-frequency hearing loss in *Espnl* null mice

To determine the role of ESPNL in hair-bundle function, we generated a knockout mouse line using the clustered regularly interspaced short palindromic repeats (CRISPR)/Cas9 nuclease technique^28^. By delivering guide RNAs (gRNAs) directed at exons 1 and 8, we generated a 25 kb deletion in the *Espnl* gene (*Espnl^Δ^*; Fig. 4f, Supplementary Fig. 9a,b). Complete loss of antibody labelling with BG35961 in the cochlea (Fig. 4g,h) indicated that this mutation was a null. We identified ∼15 additional alleles with insertions or deletions in either exons 1 or 8 (Supplementary Fig. 9c). We examined one of these (*Espnl^A^*), which has the addition of a single adenine in exon 8 and is predicted to form a truncated protein. Indeed, no immunoreactivity was detected in mice homozygous for this mutation using the BG35961 antibody.

When assessed by confocal microscopy, the overall morphology of early postnatal hair bundles appeared normal in *Espnl^Δ^*^*/*^*^Δ^* cochleas. When measured by auditory brainstem responses (ABR) at ∼1 month, *Espnl^Δ^*^*/*^*^Δ^* and *Espnl^A/Δ^* mice had high-frequency hearing loss (Fig. 4i). The tm1b allele of *Espnl* produced and phenotyped by the International Mouse Phenotyping Consortium had a very similar high-frequency hearing loss (http://www.mousephenotype.org/data/genes/MGI:2685402). Examination of bundles of P10.5 cochlear outer hair cells in basal regions, where high frequencies are encoded, showed that the shortest row (row 3) of stereocilia was mostly missing from *Espnl^Δ^*^*/*^*^Δ^* bundles (Fig. 4j,k). Outer hair cells from mid regions and the apex appeared normal, as did all inner hair cells. At P30.5, the pattern was similar, suggesting that there was no degeneration of hair cells lacking row 3 stereocilia. ESPN-1 was present at P9 stereocilia tips at similar levels in apical and basal hair bundles (Supplementary Fig. 10), suggesting that the ESPNL phenotype was independent of the presence of ESPN-1. The organization of hair bundles in *Espnl^Δ^*^*/*^*^Δ^* utricles appeared relatively normal in both striolar and extrastriolar regions, and no overt vestibular phenotype was observed.

### ESPNL is transported by MYO3A and MYO3B

**Figure 5:**
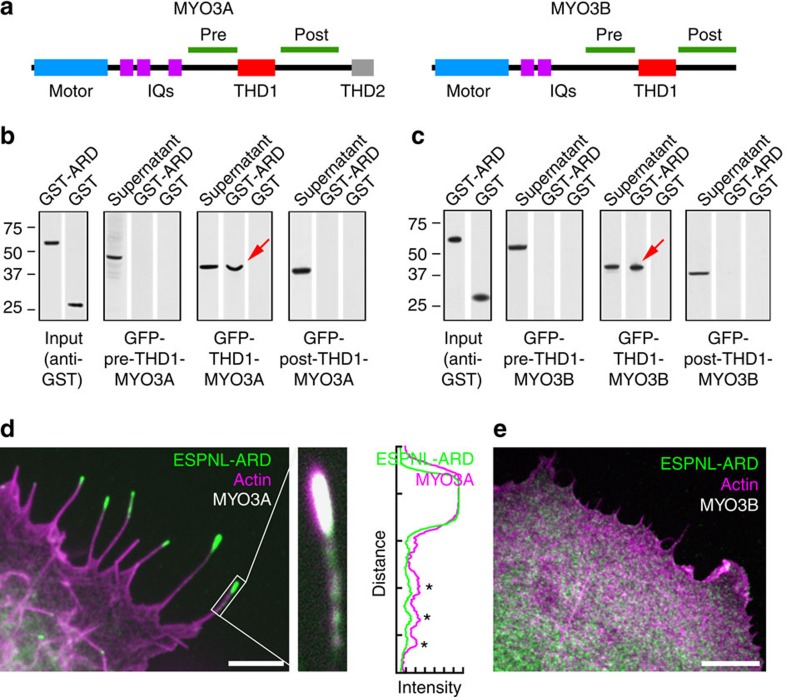
ESPNL binds myosin-III tail-homology domain 1. (**a**) Domain structure of MYO3A and MYO3B. Light blue, motor domain. Magenta, calmodulin-binding IQ domains. Red, tail homology domain 1 (THD1). Grey, tail homology domain 2 (THD2). MYO3B lacks THD2. (**b**,**c**) ESPNL ARD binds THD1 of MYO3A (**b**) and MYO3B (**c**) indicated by red arrows. GFP-tagged MYO3A-3THDI and MYO3B-THDI protein from the cell lysates bound to glutathione-agarose beads preloaded with GST-ESPNL-ARD but not beads with GST only. Pre- and post-THDI MYO3A and MYO3B constructs did not bind to GST-ESPNL-ARD beads. (**d**) (left) GFP-ESPNL-ARD is transported to filopodia tips by MYO3A (red channel, phalloidin). (centre) Magnified view of one filopodium (red channel, MYO3A). (right) Intensity profile of filopodium. ESPNL-ARD and MYO3A puncta (asterisks) may represent transport aggregates. (**e**) GFP-ESPL-ARD is not transported into filopodia by MYO3B. Scale bars, 2 μm.

The ESPN-1 ARD interacts with the tail homology domain 1 (THD1) of both MYO3A and MYO3B^13^,^14^. The ARDs of ESPN-1 and ESPNL share 54% sequence homology and are predicted to fold similarly (Fig. 4a; Supplementary Fig. 11). Using pull-down assays, we found that purified GST-tagged ESPNL-ARD bound to GFP-tagged MYO3A-THDI (Fig. 5b) or MYO3B-THDI fragments (Fig. 5c), but not to regions N- or C-terminal of the THD1. When expressed together in COS7 cells, ESPNL-ARD was transported with MYO3A to filopodia tips (Fig. 5d). By contrast, ESPNL-ARD and MYO3B remained diffuse in the cytoplasm (Fig. 5e). When full-length ESPNL was co-expressed with either MYO3A or MYO3B; however, ESPNL co-localized with each motor at filopodia tips (see Fig. 7). Thus the ARDs of ESPN-1 and ESPNL each bind to the THD1 of the myosin-III paralogs, but like with ESPN-1 (ref. 14), other domains of ESPNL enable MYO3B tip-directed motility.

### Myosin III paralogs substitute for each other in hair cells

**Figure 6:**
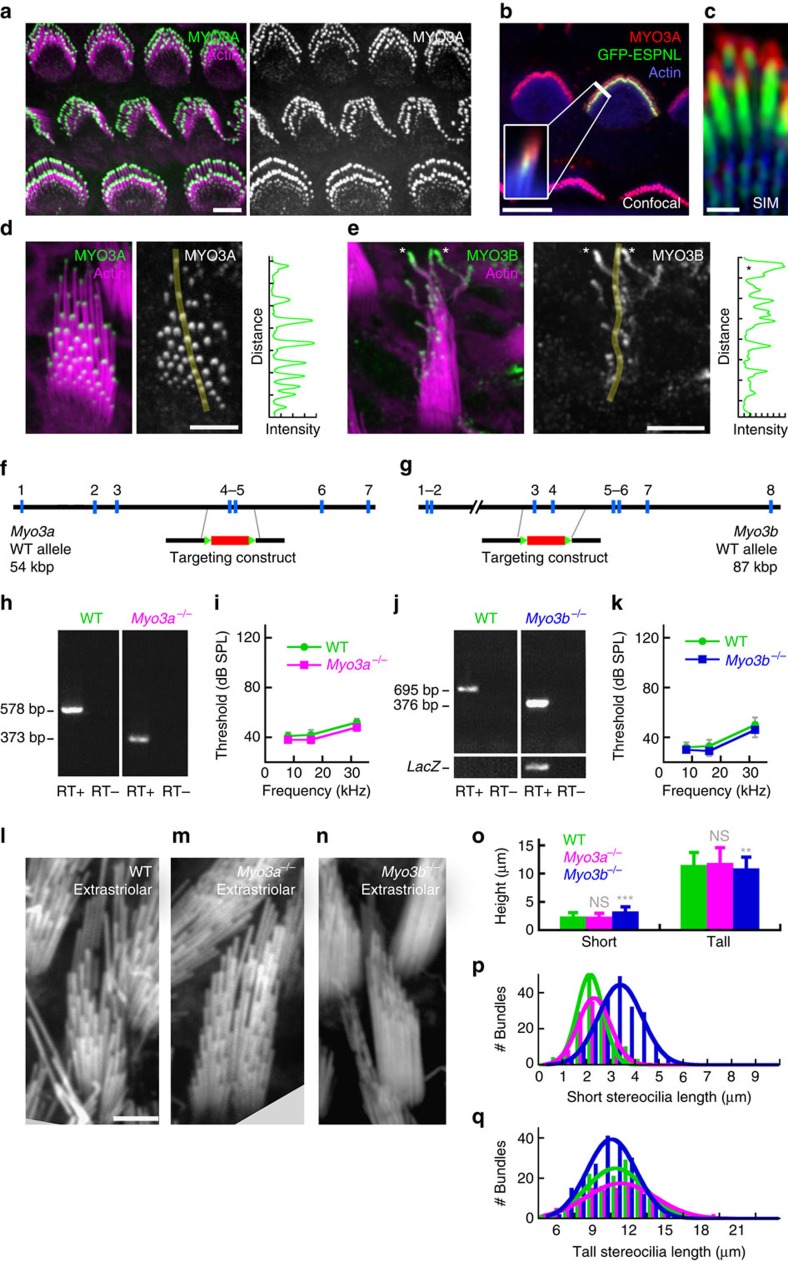
MYO3A and MYO3B separately localize in bundles but replace each other in the ear. (**a**) Immunolabelling of MYO3A (green) in rat auditory hair cells (P3); actin (magenta) labelled with phalloidin. (right) MYO3A label only. (**b**) MYO3A and ESPNL-GFP do not completely overlap in hair bundle of outer hair cell (see inset). Elongated ESPNL-GFP labelling in row 2. (**c**) SIM image of outer hair-cell stereocilia showing MYO3A cap and elongated ESPNL-GFP. (**d**) MYO3A labelling in utricle bundle. (left) MYO3A image with transect indicated. (right) Intensity profile along transect. (**e**) (left) MYO3B labelling is greatest in tallest stereocilia (asterisks). (right) Intensity profile along transect. (**f**) Representation of exons 1–7 (blue boxes) of WT mouse *Myo3a* locus. The targeting vector replaced exons 4 and 5 with a floxed neomycin-resistance gene cassette, introducing a premature stop codon in exon 6. (**g**) Exons 1–8 of the *Myo3b* locus; the targeting vector (middle) was designed to eliminate exons 3 and 4, replacing them with a neomycin-resistance gene cassette conjugated to LacZ; a premature stop codon was introduced in exon 5. (**h**) RT–PCR on inner-ear mRNA showing 578 bp amplicon from WT mouse and expected 205 bp truncated band in *Myo3a*^−/−^. (**i**) Mean ABR measurements (±s.d.) from *Myo3a*^−/−^ (*n*=29) and *Myo3a^+/−^* (*n*=27) mice at 6 months. (**j**) RT–PCR on inner-ear mRNA showing 695 bp amplicon from WT mouse and expected 319 bp truncated band from *Myo3b*^−/−^. (**k**) ABR from *Myo3b*^−/−^ (*n*=9) and *Myo3b^+/−^* (*n*=9) mice at 6 months. (**l**) WT extrastriolar bundle (phalloidin stained). (**m**) *Myo3a*^−/−^ extrastriolar bundle. (**n**) *Myo3b*^−/−^ extrastriolar bundle. (**o**) Heights of short and tall stereocilia from indicated genotypes. Mean±s.d.; *n*=148 (WT), 123 (*Myo3a*^−/−^) and 199 (*Myo3b*^−/−^). Student’s *t*-test indicated *Myo3b*^−/−^ short stereocilia lengths are different from WT at *P*=10^−21^; tall lengths are different at *P*=0.007. (**p**) Distribution of short stereocilia lengths. (**q**) Distribution of tall stereocilia lengths. Scale bars, (**a**,**b**,**d**,**e**) 5 μm; (**c**) 300 nm; and (**l**–**n**) 2 μm.

Results seen with the *Espn-1* and *Espnl* knockouts suggested that cargo interaction with each myosin-III paralog was important for hair-cell function. In the mouse utricle, MYO3A was elevated in hair cells of the striolar region (Fig. 2l), and concentrated at the distal tips of auditory (Fig. 6a–c) and vestibular (Fig. 6d) stereocilia. Although enrichment of MYO3A was largely correlated with stereocilia length^15^, higher magnification imaging in utricle bundles showed that MYO3A was low in the very shortest stereocilia, rose in intensity in the intermediate-length stereocilia, and then fell off in intensity in the longest stereocilia (Fig. 6d). By contrast, MYO3B was concentrated in the longest stereocilia, although it was also present at lower levels at the tips of other stereocilia (Figs 1m, 6e).

To examine the role of the myosin-III paralogs within stereocilia, we generated *Myo3a*^−/−^ and *Myo3b*^−/−^ mice (Fig. 6). Hair-bundle organization and structure in auditory organs of both mutant lines appeared to be normal by SEM and confocal fluorescence microscopy; moreover, hearing thresholds were normal, even at 13 months (Fig. 6i,k).

While neither *Myo3a*^−/−^ nor *Myo3b*^−/−^ mice showed any overt vestibular dysfunction, we did note a subtle defect in stereocilia-staircase step spacing in the extrastriolar regions of the utricle. Blind measurements of hair bundles imaged with confocal microscopy showed that *Myo3b*^−/−^ extrastriolar hair cells had a significantly smaller length difference between tallest and shortest stereocilia than did *Myo3a*^−/−^ or WT mice (Fig. 6l–q). This effect was reminiscent of that seen with *Espn-1*^−/−^ mice, but in *Myo3b*^−/−^ hair cells was due mostly to net elongation of short stereocilia (Fig. 6o,p). Shortening of tall stereocilia in *Myo3b*^−/−^ hair cells, while statistically significant, was less noticeable.

We next attempted to generate homozygous null *Myo3a*^−/−^ *Myo3b*^−/−^ mutants. We used several different breeding schemes to generate double-null mice (Supplementary Fig. 12). Although expecting ∼12 double knockouts from our 88 progeny, we obtained none. We also obtained no *Myo3a*^+^*^/^^−^ Myo3b*^−/−^ mice, despite expecting ∼9. Using a χ^2^-test and computing *P* values by Monte Carlo simulation^29^, the obtained genotype distribution differed from that expected by *P*<0.001. Double-null embryos presumably died during development, and a single WT allele of *Myo3a* was unable to substitute for the loss of both *Myo3b* copies.

### Filopodia elongation depends on cargo and myosin III

**Figure 7:**
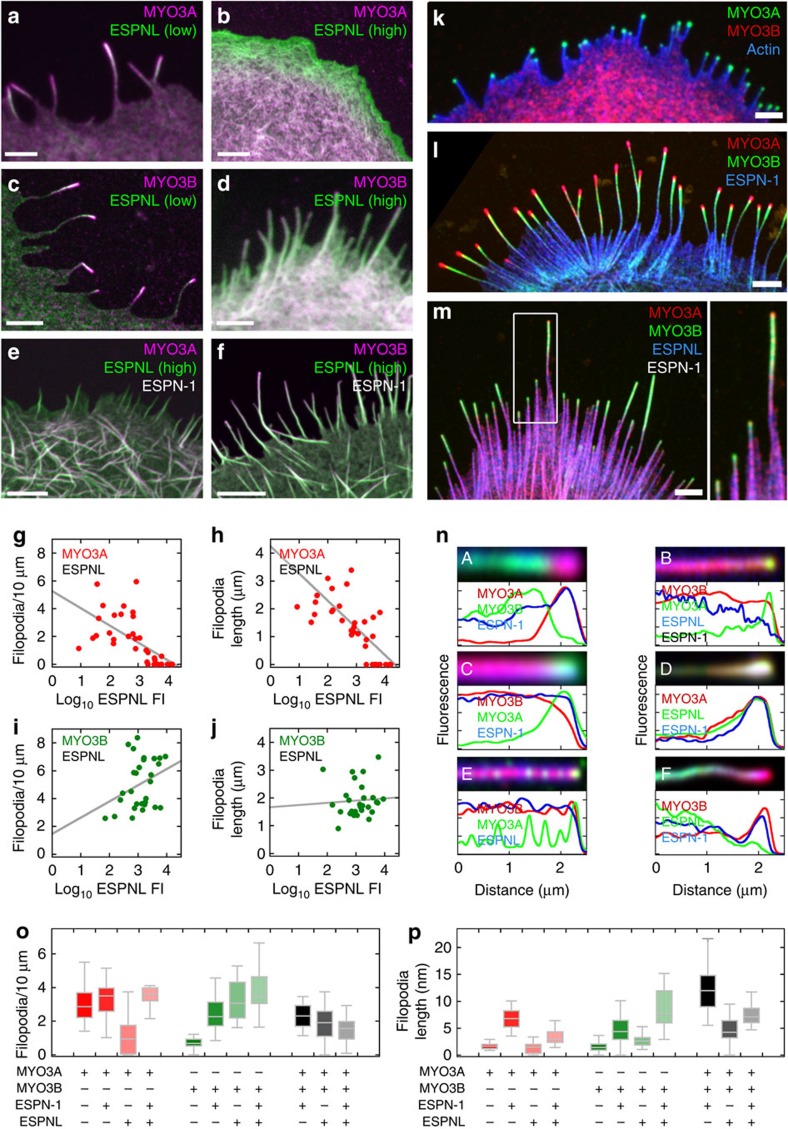
ESPN-1 and ESPNL cooperate with MYO3A and MYO3B to differentially control filopodia growth. (**a**,**b**) COS7 cells co-expressing mCherry-MYO3A (magenta) with relatively low (**a**) or high (**b**) expression of mEmerald-tagged full-length ESPNL (green). (**c**,**d**) COS7 cells co-expressing mCherry-MYO3B (magenta) with relatively low (**c**) or high (**d**) expression of mEmerald-ESPNL (green). (**e**) MYO3A is unable to target filopodia tips in the presence of ESPNL, even when co-expressed with ESPN-1. (**f**) MYO3B consistently targets filopodia tips in cells expressing both ESPNL and untagged ESPN-1. (**g**–**j**) Quantification of relationship between ESPNL fluorescence level (ESPNL Fl) and filopodia density (**g**,**i**) or length (**h**,**j**). There was an inverse relationship of filopodial number and length with ESPNL fluorescence for MYO3A (R2=0.4 and 0.5) but not MYO3B (R2<0.1 and <0.01). (**k**) COS7 cell expressing GFP-MYO3A (green) and mCherry-MYO3B (red), with actin labelled phalloidin (blue). (**l**) Filopodia are longer in the presence of MYO3A, GFP-MYO3B and ESPN1 (blue). (**m**) Filopodia are long with MYO3A, GFP-MYO3B, TagBFP2-ESPNL (blue) and untagged ESPN1. (**n**) Confocal images and relative pixel intensity profiles of single filopodia from cells transfected with the indicated constructs. Colours are identical in images and corresponding profiles. Scale in the profiles applies to the images. (**o**,**p**) Filopodia number per 10 μm of cell perimeter (**o**) and lengths (**p**) for each motor-cargo combination are presented as box plots, with upper and lower whiskers representing the range, top and bottom of the boxes representing the upper and lower 25th percentile, and the bars bisecting the boxes representing the median values (values from ∼30 COS7 cells used for each combination). Two-way analysis of variance analysis of data in these panels is reported in Supplementary Table 1. Scale bars, 2 μm.

COS7 cell filopodia provide a useful assay for the effects of the myosins and their cargos on actin-protrusion elongation. To determine how ESPN-1 and ESPNL interact with myosin-III paralogs, we expressed various combinations of tagged proteins in COS7 cells, and monitored filopodia initiation and length (Fig. 7). To simplify our analysis, we used myosin-III constructs that lacked the N-terminal kinase domain, which normally autoinhibits the myosin motor activity^30^,^31^. Consistent with previous reports^14^, MYO3A alone induced the formation of filopodia, while MYO3B did not (Fig. 7o). When co-expressed with ESPN-1, MYO3A and MYO3B each produced filopodia that were significantly longer than with the motor alone (Fig. 7p; Supplementary Table 1).

ESPNL had strikingly different effects on filopodia elongation depending on which myosin-III was co-expressed. Cells expressing MYO3A and low levels of ESPNL had filopodia with ESPNL at their tips (Fig. 7a,g); as the expression level of ESPNL increased; however, filopodia eventually disappeared (Fig. 7g) and MYO3A co-localized with actin structures in the cytoplasm (Fig. 7b). The number and mean length of filopodia in cells expressing MYO3A and ESPNL were inversely correlated with the ESPNL concentration (Fig. 7g,h). By contrast, in cells expressing MYO3B and ESPNL, co-transport of both proteins to filopodia tips was consistently observed in cells expressing either low or high levels of ESPNL (Fig. 7c,d). With MYO3B, ESPNL concentration and number or mean length of filopodia were uncorrelated (Fig. 7i,j). In both cases, the effects of ESPNL on filopodia number and length were highly significant (Supplementary Table 1).

When MYO3A was present with high ESPN-1 and low ESPNL within a filopodium, all three proteins moved together to the distal tip (Fig. 7nD). The fluorescence-intensity profile of ESPN-1 within the filopodium was shifted slightly tipward relative to MYO3A, however, and ESPNL was shifted proximally (Fig. 7nD), suggesting that the two cargos compete to bind to MYO3A. By contrast, when both cargos were expressed with MYO3B, little ESPNL was near filopodia tips, unlike ESPN-1 (Fig. 7nF).

MYO3B lacks the THDII domain of MYO3A (refs 14, 32). When we co-transfected COS7 cells with ESPNL and a hybrid protein in which THDII from MYO3A was fused onto the C-terminus of MYO3B (MYO3B+THDII), the hybrid mimicked MYO3A. In cells expressing lower levels of ESPNL, filopodia were formed and both ESPNL and MYO3B+THDII localized to their tips; by contrast, in cells expressing high levels of ESPNL, formation and elongation of filopodia containing MYO3B+THDII was reduced or abolished (Supplementary Fig. 13). The actin-binding THDII domain thus causes MYO3A to respond to the level of ESPNL much differently than MYO3B.

The number and length of filopodia that were induced by ESPN-1 and either MYO3A or MYO3B were affected differently when ESPNL was present. When high levels of ESPNL were co-expressed with MYO3A and ESPN-1, both the number and length of filopodia formed were reduced. Instead, we observed increased formation of cytoplasmic actin cables along which ESPNL and MYO3A co-localized (Fig. 7e,o,p). By contrast, ESPNL enhanced filopodia elongation by ESPN-1 and MYO3B, even at high ESPNL expression levels (Fig. 7f,o,p).

Filopodia formed in the presence of ESPN-1 and both MYO3A and MYO3B were significantly longer than those formed in the presence of ESPN-1 and only one of the myosin-III paralogs (Fig. 7l,p). ESPNL had a more modest enhancement of filopodia length in the presence of both motors, and ESPNL antagonized the lengthening seen with MYO3A, MYO3B and ESPN-1 (Fig. 7m,p). When ESPN-1 was co-expressed with MYO3A and MYO3B, ESPN-1 and MYO3A co-localized at filopodia tips and the tip-to-base gradient of MYO3A was extended^13^ (Fig. 7nA, nC). MYO3B entered filopodia when ESPN-1 was present, but was consistently proximal to MYO3A along the filopodial shaft with a very extended tip-to-base gradient (Fig. 7nA, nC).

When we co-expressed ESPNL with MYO3A and MYO3B (Fig. 7nE), the position of MYO3B became more tipward than with ESPN-1. Although exhibiting an extended comet-tail distribution pattern, MYO3B often localized more distally relative to MYO3A, frequently concentrating at filopodia tips. Conversely, MYO3A was often reduced or absent from filopodia tips, and was often only detectable towards the base of the filopodia immediately contacting the cell body. This result was consistent with the inhibition of filopodia tip-directed motility of MYO3A by ESPNL (Fig. 7b). When both cargos were co-expressed with MYO3A and MYO3B (Fig. 7nB), we observed further variations in their relative distributions within filopodia. While MYO3B was still consistently distal along the filopodium, relative to MYO3A, small amounts of MYO3A sometimes accumulated at filopodia tips (Fig. 7nB).

## Discussion

We show here that MYO3A and MYO3B, along with their actin-regulating cargos ESPN-1 and ESPNL, form a cooperative system whereby different motor-cargo combinations control the number and length of actin protrusions produced by a cell. While MYO3A and MYO3B largely substitute for each other if two wild-type alleles of either motor are present, ESPN-1 and ESPNL interact differently with the two motors and cannot replace each other fully, at least in hair cells. Most notably, the inner-ear phenotypes of *Espn-1* and *Espnl* null mice suggest that this motor-cargo system is used to control the step size of the stereocilia staircase.

We used filopodia formation in COS7 cells as a model system for understanding how cells control the number and length of cellular processes containing crosslinked parallel actin filaments, such as control of stereocilia length by hair cells. Acting within restricted compartments at distal tips, the myosin-III isoforms and their cargos control the length of actin-rich filopodia and molecular transport within them^33^. Expression in COS7 cells of MYO3A, MYO3B, ESPN-1 and ESPNL alone and in combinations allowed us to work out the intricacies of their activities and interactions. As shown previously, MYO3A alone stimulated filopodia formation, but MYO3B did not^13^,^14^; when no motors were present, ESPN-1 elongated filopodia but ESPNL did not. Combinations of motor and cargo revealed further complexities in the system. Most importantly, while ESPN-1 and ESPNL could each combine with MYO3B to elongate actin processes, MYO3A-dependent elongation was activated by ESPN-1 but inhibited by ESPNL. ESPNL also inhibited lengthening when expressed in combination with ESPN-1, either with MYO3A alone or when both motors were present.

To probe the role of the myosin-cargo system in the inner ear, we analysed a series of knockout mouse lines. While MYO3A and MYO3B are differentially expressed in regions of vestibular organs, single *Myo3a* and *Myo3b* knockouts nevertheless showed that the two motors are almost interchangeable during stereocilia development. Moreover, subtle defects in stereocilia-staircase spacing of *Myo3b*^−/−^ hair cells indicated that MYO3A cannot fully substitute for MYO3B, especially in regions like the extrastriolar utricle where little MYO3A is expressed. By contrast, there presumably was enough MYO3B in hair cells of *Myo3a*^−/−^ mice to allow them to develop their stereocilia normally; indeed, the overall level of MYO3B in utricles is much greater than that of MYO3A, suggesting MYO3B is the predominant paralog. That said, humans who are homozygous or compound heterozygote for recessive *MYO3A* mutations suffer from non-syndromic hearing loss (DFNB30) that is progressive^19^, which suggests that substitution of MYO3B for MYO3A is not completely effective in the cochlea.

Double knockouts indicated at least one *Myo3b* allele is required for embryonic development; no *Myo3a*^−/−^ *Myo3b*^−/−^ or *Myo3a^+/−^ Myo3b*^−/−^ mice were born from crosses that should have generated substantial numbers of them. These results suggest that these motors are required in other tissues, in addition to inner ear^14^,^15^ and retina^34^, a prediction that is supported by examining their tissue expression using the Illumina Body Map (http://www.ebi.ac.uk/gxa/experiments/E-MTAB-513).

Myosin-III motors require ESPN-1 or ESPNL to function in hair cells. ESPNL was the most prominent developmentally regulated hair-bundle protein in our proteomics experiments, and was highly enriched in bundles. Like ESPN, ESPNL both binds to actin and has a proline-rich domain that may bind profilin-actin; this domain may allow ESPNL to concentrate actin monomers and contribute to stereocilia elongation^16^. Indeed, ESPNL allows filopodia to be formed with MYO3B. When low levels of ESPNL are present, MYO3A-dependent filopodia can be formed; by contrast, high levels of ESPNL inhibit filopodia elongation when MYO3A is present.

Like that of *Espn-1*^−/−^ mice, the phenotype of *Espnl*^−/−^ mice was apparent in only a subset of hair cells; while there were no apparent vestibular functional defects, sensitivity towards high-frequency auditory stimuli was drastically reduced. Consistent with high-frequency hearing loss, *Espnl*^−/−^ mice had altered bundles of outer hair cells—largely missing stereocilia row 3—only at the cochlea’s basal end, which encodes high frequencies. While the loss of one stereocilia row should decrease the total amount of transduction current by ∼50%, auditory sensitivity may drop by far more than that given the special role of outer hair cells in cochlear amplification^35^. Given the greater ESPNL abundance in the second row, loss of the third row was puzzling; ESPNL activity in short stereocilia could, however, prevent stereocilia destabilization that is predicted to occur below a certain length^7^. Regardless, we presume that *Espnl*^−/−^ mice have a restricted phenotype because ESPN-1 can substitute in activating myosin-III molecules in most hair cells.

In COS7 cells, ESPN-1 stimulated filopodia initiation and lengthening regardless of whether MYO3A, MYO3B or both motors were present. In hair cells, ESPN-1 accounted for <2% of the total ESPN protein but was responsible for a key subset of functions carried out by ESPN. The morphology and function of hair bundles of *Espn-1*^−/−^ mice differed from those of *jerker* mutant mice, which lack all ESPN isoforms and are profoundly deaf with severe vestibular dysfunction^21^,^36^,^37^. Homozygous *jerker* stereocilia completed neither diameter growth nor elongation during bundle formation, but instead bent, shortened and disappeared^36^. In contrast, stereocilia in auditory and vestibular hair cells of *Espn-1*^−/−^ mice persisted through adulthood, albeit with a altered phenotype in extrastriolar hair cells.

While stereocilia diameter was reduced in extrastriolar hair cells of *Espn-1*^−/−^ mice, consistent with the observation that ESPN-1 controls the size of actin bundles^18^, near-complete elimination of the stereocilia staircase in *Espn-1*^−/−^ extrastriolar regions revealed the principal role of the myosin-cargo system. Why was the phenotype constrained to a single type of hair cell? Only one myosin-III (MYO3B) was expressed in extrastriolar hair cells at substantial levels and ESPNL was largely absent. Cell-culture experiments showed that MYO3B required ESPN-1 or ESPNL for transport to distal ends of actin protrusions^14^, and MYO3B was indeed absent from tips of extrastriolar *Espn-1*^−/−^ stereocilia. The stereocilia of extrastriolar hair cells of *Espn-1*^−/−^ mice thus uniquely lacked activity of both myosin-III and its actin-regulatory cargos, while other *Espn-1*^−/−^ hair cells presumably retained ESPNL-dependent activation of MYO3B. The more subtle defect in staircase formation observed in *Myo3b*^−/−^ mice reinforced this interpretation; a small amount of MYO3A present in extrastriolar hair cells of *Myo3b*^−/−^ mice presumably could partially control stereocilia spacing. By contrast, in other single knockouts we examined, where MYO3A or ESPNL were missing, each hair cell expressed detectable levels of both a myosin-III paralog and either ESPN-1 or ESPNL. For example, some hair cells of *Espnl*^−/−^ mice still express ESPN-1, which can activate either MYO3A or MYO3B.

Although the myosin III-cargo system can lead to massive outgrowth of filopodia in COS7 cells, the *Espn-1*^−/−^ extrastriolar phenotype showed that in hair cells, a critical function of this system is to instead control the relative step size of the stereocilia staircase. A comprehensive model of actin-process elongation explains the formation of staircase spacing by invoking gradients of actin-polymerization activators or physical properties at the apical surface^6^,^7^. Because multiple mechanisms control stereocilia length^4^,^9^,^38^,^39^,^40^, and the total actin content seems to be fixed^41^, the activity of the myosin III-cargo system must be proportionally constrained to differentially activate actin elongation in successive rows of stereocilia, so that the shortest rows have the least activation and the longest rows have the most. Indeed, the length dependence of MYO3A and MYO3B localization in stereocilia, as well as the phenotype seen in *Myo3b*^−/−^ hair cells, both consistent with such a mechanism. Moreover, given the inhibitory activity of elevated levels of ESPNL on motor-cargo complexes that include MYO3A, the large amounts of ESPNL at tips of row 2 of cochlear inner hair cells may serve to disproportionately suppress growth of this stereocilia row, generating the much smaller rows 2 and 3 step spacing than that of rows 1 and 2. Likewise, later in development ESPNL accumulates at the tips of row 3 stereocilia, presumably suppressing elongation there.

The functional significance of staircase step spacing is not fully understood. A small intrinsic gradation in stereocilia height seems to be an essential part of a bundle^42^, and may be set up at the earliest stage of bundle development^43^; a further increase in step spacing occurs after the minimal spacing is established^3^,^36^. While step spacing is thought to have little effect on hair-bundle stiffness^44^, this distance could control the diffusion of Ca^2+^ to the upper end of the tip link in vestibular hair cells and perhaps the rate of slow adaptation^40^,^45^.

The restricted phenotypes in *Myo3a*^−/−^, *Myo3b*^−/−^, *Espn-1*^−/−^ and *Espnl*^−/−^ mice reinforce the suggestion that robust compensatory mechanisms control stereocilia length. For example, ESPNL enhances outgrowth of MYO3B-dependent actin processes, yet inhibits those dependent on MYO3A or motor mixtures. When hair cells lack ESPN-1 or MYO3A, MYO3B-ESPNL complexes apparently rescue control of stereocilia length. Stereocilia must maintain the staircase for a lifetime, even though actin at tips is dynamic^38^,^39^,^42^,^46^,^47^,^48^, suggesting that considerable redundancy must be built in. Elimination by P10 of the ectopic long microvilli present in *Espn-1*^−/−^ auditory hair cells may be an example of the hair cell’s ability to overcome transient misregulation of actin-protrusion length. The myosin-III plus cargo system operates using selective protein expression, targeting of individual motors to specific actin protrusions, strong stimulation of actin elongation by ESPN-1, and differential stimulation of elongation by ESPNL depending on motor expression; two motors and two cargos allows for combinatorial control. Endogenous regulation likely involves auto- and inter-molecular phosphorylation by the myosin-III kinase domains^30^,^31^ or interaction with other cellular components^49^. Together this system allows the cell to precisely tune individual actin protrusions’ lengths, which is essential for the normal formation of elaborate cellular architecture like hair-cell stereocilia.

## Methods

### Animals

The care and use of animals for the experiments described conformed to NIH guidelines and were approved by the Institutional Animal Care and Use Committees at the National Institute on Deafness and Other Communication Disorders (NIDCD ACUC), Oregon Health and Science University (OHSU IACUC) and the University of Nebraska-Lincoln (UNL IACUC). In all experiments, a mixture of male and female mice were used. No gender-specific effects were noted.

### Knockout mice

Standard methods were used to generate mouse founders carrying deletions of exons 4 and 5 in *Myo3a* allele, and of exon 1 in *Espn* allele at the Animal Transgenic Facility of the National Eye Institute (Bethesda, MD). Exons 3 and 4 were targeted in *Myo3b*, which was carried out by the Texas A&M Institute for Genomic Medicine (College Station, TX). Final targeting vectors were transfected into mouse ES cells (SV129) by electroporation. After selection based on drug resistance, correctly targeted ES cell clones were screened by long-range PCR, verified by Southern blotting, and microinjected into blastocysts. At least five chimeric mice were crossed with C57BL6/J partners for germline transmission of the modified allele. Founders for each genetically modified colony were selected by long-range PCR genotyping and were backcrossed onto the C57B6/J background for five generations or more before phenotypic evaluation of homozygote mutants.

For the *Espnl* knockout with CRISPR, gRNAs targeted to *Espnl* exons 1 and 8 were selected using the CRISPR web based tool (http://crispr.mit.edu/). The gRNA sequences (exon 1, 5′-GTGCATCATGCCACCCGGGC-3′; exon 8, 5′-CCGGCCACGCTCGTCCTGTG-3′) were individually cloned into the DR274 plasmid (Addgene #42250) and transcribed using the MegaScript kit (LifeTechnologies). gRNAs were purified using the NucleoSpin miRNA (Macherey-Nagal) and quantified. Zygotes were injected with a cocktail containing 30 ng μl^−1^ of each gRNA and 110 ng μl^−1^ of Cas9 mRNA (Trilink) and implanted into pseudopregnant females. All founders were screened for mutations in *Espnl* exon 1, exon 8 and any intervening deletions between these two exons. We characterized two alleles in depth: c.135_1301del1165 (forming protein p.A46Tfs14; referred to as *Espnl^Δ^*), and c.1298_1299insA, in which a single A is inserted in exon 8 (forming protein p.Q433Qfs10; referred to as *Espnl^A^*). All genes with predicted off-target sites (exon 1: *AW551984*, *C1ql1*, *Cyp26c1*, *Olfr523*, *Pdc4c*, *Pomt2* and *Slc12a7*; exon 8: *Acss2*, *Elf4*, *Krt79*, *Lrrc30*, *Ncoa4* and *Phf2*) were sequenced in our founder mice; all off-target sites were WT.

### Antibodies

Affinity-purified rabbit polyclonal antibodies specific for mouse ESPN-1 (PB538/539), MYO3A (PB638) and MYO3B (PB791) have been previously described^13^,^14^,^15^. In some experiments, we used a rabbit polyclonal antibody against a C-terminal region of human ESPNL (ab170747; Abcam, Cambridge, MA). In addition, Genemed Synthesis (San Antonio, TX) generated for us a rabbit antiserum directed to a mixture of four peptides from the C-terminal half of ESPNL: BG35959, HWKKSAYTPALRTAACRT (residues 748–763); BG35960, [C]MAHVPARQLRRLSRR (814–828); BG35961, CDLPAEERKMRHLL (868–880); and BG35962, CFEVFEHLGAHGWEAVRAFHK (882–901). To purify selective antibodies, this antiserum was run separately over individual peptides conjugated to SulfoLink (Life Technologies); BG35960 and BG35961 were found to give the best signal-to-noise ratio.

### Expression plasmids

Because the N-terminal kinase domain of class III myosins has been shown to downregulate motor activity^30^,^50^, all MYO3A and MYO3B constructs used in our study lacked this domain. GFP-MYO3A, GFP-MYO3A-pre-THDI, GFP-MYO3A-THDI, GFP-MYO3A-post-THDI and GFP-ESPN-1, constructs used have been previously described^13^. Likewise, GFP-MYO3B, GFP-MYO3B-pre-THDI, GFP-MYO3B-THDI, GFP-MYO3B-post-THDI, mCherry-MYO3B and hybrid mCherry-MYO3B:THDII constructs also have been previously described^14^. The mCherry-MYO3A construct used was described in ref. 30. mEmerald- and mCherry-tagged ESPNL were generated by fusing mEmerald or mCherry, respectively, to the N- or C-terminus of ESPNL in N3-Clontech vectors. Briefly, for N-terminally tagged ESPNL, the following primers were used to PCR-amplify mouse *Espnl* and create NheI and BamHI restriction sites: NheI forward:5′-GTCAGATCCGCTAGCACCGCCACCATGGCTGCAGTGACCATGTCCGTGTCT-3′; and BamHI reverse:5′-CCTGTACGGATCCGCGCTACCACTGGCTGCGCTTGCTCCACCGCTGCTTTGGGGTGTGGCAGGGGGCG-3′. The PCR product was digested and ligated into a similarly cut mCherry-C1 or mEmerald-C1 cloning vector to yield a fusion of mCherry or mEmerald, respectively, with Espnl separated by an 18 amino acid linker (mCherry-18-ESPNL or mEmerald-18-ESPNL). To label the ESPNL C-terminus, PCR-amplified Espnl was ligated into a similarly cut mCherry-N1 or mEmerald-N1 vector to yield a fusion of ESPNL-18-mCherry or ESPNL-18-mEmerald, respectively. All DNA for transfection was prepared using the Plasmid Maxi kit (QIAGEN, Valencia, CA) and characterized by transfection in HeLa cells (CCL2 line; ATCC, Manassas, VA) using Effectene (QIAGEN) followed by observation under wide-field fluorescence illumination to ensure proper localization (ET-GFP and ET-DsRed filter sets; Chroma, Rockingham, VT). The sequences for all vectors were confirmed using Big Dye technology by the Florida State University DNA Sequencing Laboratory in the Department of Biological Science. The GST-ESPNL construct was generated by fusing the GST tag to the N-terminus of ESPNL housed in a N1-Clontech vector. The following primers were used to PCR-amplify the GST tag and create AgeI and BspEI restriction sites: AgeI forward: 5′-GCGCTACCGGTCGCCACCATGTCCCCTATACTAGGTTATTGGAAAATTAAGGGCCTTGTGCAAC-3′; BspEI reverse: 5′-TCTGAGTCCGGAACGCGGAACCAGATCCGATTTTGGAGGATGGTC-3′. The PCR product was digested and ligated into a similarly cut ESPNL-N1 cloning vector to yield a fusion of GST with ESPNL separated by a 22 amino acid linker (GST-22-ESPNL).

### Immunofluorescence and light microscopy

For experiments conducted at the NIDCD, mice were killed and temporal bones were dissected; they were then perfused through the round window with 4% formaldehyde in phosphate buffered saline (PBS; pH 7.4) and fixed for 20 min at room temperature. Inner-ear epithelia were micro-dissected in PBS before permeabilization with 0.5% Triton X-100 for 30 min, and blocking overnight at 4 °C in filtered 4% bovine serum albumin (BSA) in PBS. In the case of PB791, tissue was treated for antigen retrieval before blocking, by incubating in 1 mM TRIS-EDTA buffer pH 9, at 60 °C for 1 h. To process tissue for immunofluorescence^15^, it was first incubated with primary antibody (1 μg ml^−1^) for 2 h, rinsed with PBS three times, stained with goat anti-rabbit secondary antibodies (Molecular Probes) conjugated with AlexaFluor 488 (A-11001) or Alexa Fluor 568 (A-11011) diluted 1:600 in PBS-BSA for 1 h, and counterstained with AlexaFluor-568 phalloidin (Molecular Probes, A-12380), AlexaFluor-488 phalloidin (Molecular Probes, A-12379) or AlexaFluor-647 phalloidin (Molecular Probes, A-22287) diluted 1:600 in PBS-BSA. Tissue was then rinsed with PBS three times, and mounted using ProLong Gold Antifade reagent (Invitrogen). Confocal imaging of both tissue as well as cell-culture experiments was accomplished using a TiE inverted fluorescence microscope (Nikon Instruments) equipped with either (1) a spinning disk confocal head (Perkin-Elmer), DU-888 EMCCD (Andor) and Apo TIRF 1.49 NA objective; or (2) a swept-field confocal scan head (Prairie Technologies), DU-897 EMCCD (Andor), and × 100 Plan Apo 1.45 NA objective. All image acquisition was managed through NIS-Elements software (Nikon Instruments).

For experiments conducted at OHSU, mice were killed and temporal bones were dissected; the inner-ear tissue was immersed in 4% formaldehyde for 45 min. Following three washes in 1 × PBS the organ of Corti was dissected from the inner ear and further fixed in 4% formaldehyde for 15 min. Tissue was washed three times in 1 × PBS and permeabilized in 0.2% Triton X-100, 1.0% normal donkey serum and 0.1% BSA for 15 min. Tissue was washed and incubated in primary antibody (0.4 μg ml^−1^, diluted in block) overnight at 4°. Tissue was washed, then incubated for 3 h in block containing goat anti-rabbit Alexa Fluor 488 (Invitrogen, catalog #A21206; 4 μg ml^−1^) and CF633 phalloidin (Biotium, catalog #00046; 1:250). Lastly, tissue was washed in 1 × PBS and mounted in Everbrite (Biotium). Samples were imaged on a Zeiss LSM 710 fitted with a × 63, 1.4 NA objective. Tissue examined by SIM was processed identically to that for confocal microscopy. SIM images were acquired on the Zeiss Elyra PS.1 and processed using the Carl Zeiss Zen 2012 (black edition, 64 bit software) version 8.0 with the default settings.

### Scanning electron microscopy

For experiments conducted at the NIDCD, after removal of the inner ear from experimental mice, a small hole was made at the top of the cochlea with the tip of a fine forceps, and the inner ear was gently flushed with ∼0.3 ml of 2.5% glutaraldehyde fixative solution for 2 h at room temperature. The membranous labyrinth containing the cochlea and vestibular end organs was removed by dissection. Specimens were processed using three 1 h incubations with 1% (w/v) OsO_4_, alternated with two 1 h incubations in 1% tannic acid (w/v). The specimens were then dehydrated with a graded ethanol series and critical point dried using liquid CO_2_ as the transitional fluid. Samples were sputter coated with carbon and platinum and viewed using a Hitachi S-4800 field-emission scanning electron microscope operated at 5 kV.

For experiments conducted at the OHSU, mouse temporal bones were removed and submerged in 4% paraformaldehyde. The inner ear was dissected from the temporal bone and forceps were used to make a small hole at the apex of the cochlea so fixative could fully penetrate the tissue. Additional dissection was performed to expose the sensory epithelium and the tectorial membrane was manually removed using fine forceps. The tissue was transferred to a solution containing 2.5% glutaraldehyde and 0.15 M cacodylate buffer at pH 7.4. Tissue was further processed using a osmium-thiocarbohydrazide^51^ method (OTOTO), which alternated three osmium (OsO_4_) incubations, each for 1 h, with two 1% thiocarbohydrazide incubations for 20 min. Tissue was dehydrated with an ethanol series and critical pointed dried using liquid CO_2_. Samples were mounted on stubs and imaged using a FEI Sirion XL30 FEG field-emission scanning electron microscope operated at 5 kV.

### Culture and biolistic transfection of rat inner-ear tissue

Organ of Corti and vestibular tissues were dissected from postnatal days 0–2 rats and attached to collagen-coated coverslips. Cultures were maintained in DMEM/F12 (Invitrogen) with 5% fetal bovine serum and ampicillin (1.5 μg ml^−1^; Sigma) and maintained at 37 °C and 5% CO_2_. For transfections, 50 μg of DNA were precipitated onto 25 mg of 1-μm gold particles and loaded into the Helios Gene Gun cartridges (BioRad). Tissue explants were transfected with the gene gun set at 95 psi of helium and maintained in culture for 18–48 h. Samples were fixed and counterstained for confocal microscope imaging as described above.

### *In utero* electroporation of mouse inner-ear tissue

C57BL/6 males were crossed with CD1 females to generate embryos for transuterine microinjection. For *in utero* injection and electroporation, pregnant females with E11.5 embryos were anaesthetized with 60–65 μg g^−1^ body weight of Nembutal in a solution containing 20.8 mg ml−1 MgSO_4_, 10% ethanol and 40% propylene glycol. A ventral laparotomy was performed to expose the uterine horn and embryos were visualized and positioned using a low-intensity halogen light. A microinjection pipette was backfilled with concentrated plasmid DNA (>3 μg) and secured in place in pipette holder coupled to a Picospritzer III microinjector. The pipette was aligned and inserted into the otocyst; compressed nitrogen was used to deliver the inoculum. Following the injection, a square wave electroporator (Protech International CUY21SC) was used to deliver a pulse of 60–100 mA to the injected embryo. Once the transuterine and electroporation steps were complete, the abdominal wall was closed using an absorbable suture. Females were monitored 60 min postoperatively and once per 24 h until birth. Immunocytochemistry was carried out as described above. For images with ESPNL-GFP alone, tissues were co-stained with CF633 phalloidin (1:250). For images with ESPNL-GFP and co-staining for MYO3A, we used the 3THDII antibody^32^ at 1:500. The secondary antibody was goat anti-rabbit Alexa Fluor 647 (Invitrogen, catalog #A31573; 4 μg ml^−1^); co-staining used TRITC phalloidin (Sigma, catalog #P1951, 1:250).

### Vestibular-evoked potential measurements

These experiments were carried out at the University of Nebraska-Lincoln. Animals were anaesthetized with ketamine:xylazine (18 and 2 mg ml^−1^), 7 μl per gram body weight, injected intraperitoneally. Core body temperature was maintained at 37.0±0.2 °C (mean±range) using a homoeothermic heating pad system (FHC Inc., Bowdoin, ME). Linear acceleration pulses (17 pulses s^−1^, 2 ms duration) ranging from +6 to −18 dB re: 1.0 g ms (where 1 g=9.8 m s^−2^), adjusted in 3 dB steps, were presented to the head in the naso-occipital axis. Stimuli were delivered using a non-invasive head clip that coupled the head to a mechanical shaker (Model ET-132–203, Labworks Inc., Costa Mesa, CA). Subcutaneous electrodes were placed at the nuchal crest, posterior to the right pinna, and at the hip for the non-inverting, inverting and ground electrodes, respectively. Electroencephalographic activity was amplified (200,000 ×), filtered (300–3,000 Hz) and digitized (1,024 points at 10 μs per point). Two hundred and fifty-six primary responses were averaged and replicated for each VsEP waveform. A VsEP intensity series was collected beginning at the maximum stimulus level (that is, +6 dB re: 1.0 g ms^−1^) with and without acoustic masking (50–50,000 Hz forward masker at 90 dB SPL), and then descending in 3 dB steps until no response was visible. Thresholds (measured in dB re:1.0 g ms−1), peak latencies, and peak to peak amplitudes were measured and analysed using *t*-tests with alpha at 0.05.

### Auditory brainstem response threshold

ABR thresholds for *Myo3a*, *Myo3b* and *Espn-1* mutant mice were measured at the NIDCD^52^. Avertin (0.015 ml g^−1^ body weight) was injected intraperitoneally for anaesthesia before recording. A sound-attenuated chamber using an auditory-evoked potential diagnostic system, calibrated by the manufacturer (Intelligent Hearing Systems, Miami, FL, USA), was used for ABR recordings. Averaged responses were recorded using three subdermal needle electrodes placed at the forehead and mastoid locations. Alternating polarity click and tone-burst stimuli of 47 μs and 5 ms duration, respectively, were used as stimuli. Presentation number ranged from 128 to 1,024 depending on signal-to-noise ratio; stimulus intensities producing suprathreshold responses were initially decreased in 10 dB steps, then by 5 dB steps at lower intensities to determine the response threshold. When no waveform was observed at the highest stimulus level (90 dB SPL), the threshold was considered to be 95 dB SPL for subsequent analyses.

ABR thresholds for *Espnl* mutant mice were measured at OHSU. The animals were anaesthetized with xylazine (10 mg kg^−1^, i.m., IVX; Animal Health Inc., Greeley, CO) and ketamine (40 mg kg^−1^, i.m.; Hospira, Inc., Lake Forest, IL), and placed on a heating pad in a sound-isolated chamber. An operating microscope was used to examine the external ear canal and tympanic membrane, ensuring the ear canal was free of wax and that there was no canal deformity, inflammation of the tympanic membrane or effusion in the middle ear. Needle electrodes were placed subcutaneously at the vertex and at the shoulder of the test ear side. A closed-tube sound-delivery system sealed into the ear canal was used to stimulate each ear separately, delivering tone bursts with a 1 ms rise time at 4, 8, 16 and 32 kHz; the tone-burst stimulus intensity was increased in steps of 5 dB. ABR thresholds, defined as an evoked response of 0.2 μV from the electrodes, were obtained separately for each ear.

### Shotgun mass spectrometry of purified hair bundles

Hair bundles were purified from either P4 to P6 or P21 to P25 mouse utricles as described^53^. For shotgun experiments, four independent preparations of 100 ear-equivalents of bundles were used for each of the two ages. In addition, four preparations of 10 utricles were obtained for each age. Trypsin-digested peptides were prepared by short SDS–polyacrylamide gel electrophoresis runs and in-gel digests and were analysed using a Thermo Electron Orbitrap Velos ETD mass spectrometer^10^,^25^. For 100 utricle ear-equivalents, NuPAGE LDS sample buffer (Invitrogen) at 1.2 × dilution with 50 mM dithiothreitol was added to a final volume of ∼50 μl; the samples were heated to 65 °C for 15 min, followed by 5 min at 95 °C. By running proteins ∼1 cm into a NuPAGE 4–12% Bis-Tris gel, proteins were separated from contaminants. After washing with water, gels were stained for 5 h with Imperial Protein Stain at room temperature (Thermo Scientific). Gels were rinsed, then, 1 cm of gel containing bundle proteins was manually sliced into six pieces, each of which was processed separately in individual siliconized tubes. Gel slices were washed with 200 μl high-performance liquid chromatography (HPLC)-grade water (vortexed 30 s), 200 μl of 50% 50 mM NH4HCO3/50% MeOH (vortexed 1 min), 200 μl of 50% 50 mM NH_4_HCO_3_/50% acetonitrile (vortexed 5 min) and 200 μl of 100% acetonitrile (vortexed 30 s). The remaining solution was removed and the gel pieces were dried briefly. Gel pieces were rehydrated with 100 μl of freshly made 25 mM DTT in 50 mM NH_4_HCO_3_, then were incubated for 20 min at 56 °C. After discarding the supernatant, 100 μl of 55 mM iodoacetamide in 50 mM NH_4_HCO_3_ was added for 20 min at room temperature in the dark. The supernatant was discarded and the gel pieces were washed twice with 400 μl HPLC-grade water. The washes were discarded and 200 μl 50% 50 mM NH_4_HCO_3_/50% acetonitrile was added (vortexed 5 min). This wash was discarded and 200 μl 100% acetonitrile was added (vortexed 1 min). The supernatant was discarded and samples were dried in the SpeedVac for 2–3 min. A 1% solution of was prepared by adding 100 μl of 50 mM NH_4_HCO_3_ to the stock aliquot with swirling to mix; this ProteaseMAX solution was kept on ice. A 1.5 ml aliquot of 0.01% ProteaseMAX/6 ng μl^−1^ trypsin was made by adding 15 μl 1% ProteaseMAX (in 50 mM NH_4_HCO_3_) to 1,440 μl of 50 mM NH_4_HCO_3_; the solution was mixed, then 45 μl of a 200 ng μl^−1^ trypsin stock (Sigma-Aldrich T6567 proteomics grade, from porcine pancreas, dimethylated), diluted fresh in 50 mM NH_4_HCO_3_, was added. To each gel piece, 30 μl of the 0.01% ProteaseMAX/6 ng μl^−1^ trypsin solution was added and incubated for 30 min at 4 °C. Gel pieces were overlaid with 20 μl of the 0.01% ProteaseMAX solution to maintain them fully submerged. Trypsin digestion was allowed to proceed for 3 h at 37 °C. The digest solution was transferred to new tubes (25–40 μl of liquid from each tube); 30 μl of 2.5% trifluoroacetic acid (in HPLC-grade water) was added to gel pieces (vortexed 15 min). The solution was removed and combined with the initial digest solution. The solution was vortexed and then the combined solution was centrifuged for 10 min at 14,000 r.p.m. in a microcentrifuge. The supernatant was transferred to 0.45 μm filter tubes (Millipore Ultrafree centrifugal filters, #UFC0HV00); samples were spun 5 min at 4,000 r.p.m. All samples were dried in the SpeedVac until virtually all solution was evaporated (∼2 h), then were stored at −80 °C before mass spectrometry.

MaxQuant version 1.4.1.2 was used with the Andromeda search engine to analyse the data^54^,^55^; relative iBAQ values were calculated for each protein by dividing the iBAQ for a protein by the sum of all iBAQ values, excluding contaminants, for a single combined run of six gel pieces^56^. The data are available via ProteomeXchange (http://www.proteomexchange.org) with identifier PXD002167, and technical aspects of the experiments were discussed and validated elsewhere^25^.

### Targeted mass spectrometry of purified hair bundles

We used PRM to measure actin (summed isoforms), MYO3A, MYO3B, ESPN, ESPN-1 and ESPNL peptides from four preparations from each age (P4–P6, P21–P25), each of 13–14 ear-equivalents of hair bundles. In-solution tryptic digests of the samples were prepared using an enhanced filter-aided sample preparation (eFASP) method^57^. Proteins were digested in the filter unit in 100 μl digestion buffer with 200 ng sequencing-grade modified trypsin (Promega) at 37 °C for 12–16 h. Peptides were isolated by centrifugation and were extracted with ethyl acetate to remove remaining deoxycholic acid^57^. Three unique peptides for each protein of interest were chosen for isolation based on previous data-dependent discovery data or from online peptide databases (www.peptideatlas.org, www.thegpm.org). Synthetic stable-isotope labelled peptides (SpikeTides-TQL) corresponding to mouse protein sequences (actin, EITALAPSTMK, AGFAGDDAPR; MYO3A, DTFPTDIVLLLR, FTSSGAVVGAQISEYLLEK, VSVVTQNAPLGNLER; MYO3B, ALQFSQDR, ILQVNSLVEAFGNAR, NRDTLPADVVVVLR; ESPN, LAPWQR, LASLPAWR; ESPN-1, DNSGATVLHLAAR, YLVEEVALPAVSR, YLVQECSADPHLR; ESPNL, CQEYESELGR and EIQECGVSVR) were obtained from JPT Peptide Technologies (www.jpt.com, Berlin, Germany) and used as internal standards; any cysteine residues were substituted by carbamoylmethylated cysteines during synthesis. The following amounts of each peptide were added (spiked in) along with the trypsin solution before digestion of each sample: actin standards, 500 fmol; ESPN, 10 fmol; ESPN1, ESPNL, MYO3A and MYO3B peptides, each 1 fmol. Calibration curves were run for all peptides by adding four different amounts of each peptide, centred around the amount spiked into the sample, to four mouse utricular lysate samples (0.5 ear-equivalents), prepared in the same way as the bundle samples. Heavy and endogenous forms of each peptide were monitored by PRM.

Peptide samples were analysed with an Orbitrap Fusion Tribrid mass spectrometer (Thermo Scientific) coupled to a Thermo/Dionex Ultimate 3000 Rapid Separation UPLC system and EasySpray nanosource. Samples were loaded onto an Acclaim PepMap C18, 5 μm particle, 100 μm × 2 cm trap using a 5 μl min^−1^ flow rate and then separated on a EasySpray PepMap RSLC, C18, 2 μm particle, 75 μm × 25 cm column at a 300 nl min^−1^ flow rate. Solvent A was water and solvent B was acetonitrile, each containing 0.1% (v/v) formic acid. After loading at 2% B for 5 min, peptides were separated using a 55 min gradient from 7.5 to 30% B, 10 min gradient from 30 to 90% B and 6 min gradient at 90% B, followed by a 19 min re-equilibration at 2% B. Peptides were analysed using the targeted MS2 mode of the Xcalibur software in which the doubly or triply charged precursor ion corresponding to each peptide was isolated in the quadrupole, fragmented by HCD and full *m*/*z* 350–1,600 scans of fragment ions at 30,000 resolution collected in the Orbitrap. Targeted MS2 parameters included an isolation width of 2 *m/z* for each precursor of interest, collision energy of 30%, AGC target of 5 × 104, maximum ion injection time of 100 ms, spray voltage of 2,400 V and ion transfer temperature of 275 °C. No more than 75 precursors were targeted in each run and no scheduling was used. Precursor isolation lists for all peptides of interest were exported from the software package Skyline (http://proteome.gs.washington.edu/software/skyline/) and imported into the Orbitrap control software.

Skyline was also used to analyse targeted MS/MS data. Chromatographic and spectral data from the RAW files were loaded into Skyline and manually analysed to determine fragment ion peaks corresponding to each peptide. RAW files were also processed using Proteome Discoverer (Thermo Scientific) software in order to match MS/MS spectra to an Ensembl spectral database using Sequest HT. Fragment ion peaks that co-eluted with the fragment ion peaks for the corresponding heavy peptide were chosen for analysis. The type and proportion of daughter ions contributing to the peptide peak were required to match that of the heavy peptide peak. In addition, one or more spectra within the light or heavy peptide peak were matched to the correct peptide sequence within the spectral database. If spectra within a specific sample were not identified, then we required both that the retention time of the chosen peak be within 2 min of the retention time of an identified peak for that peptide from another sample, as well as that the type of daughter ions contributing to the peak must match the identified peptide peak from another sample. Chromatographic peak areas from all detected fragment ions for the light and heavy version of each peptide were integrated and summed, and then the peak area ratio between the light and heavy peptides was calculated. This ratio was multiplied by the amount of spiked heavy peptide to give an fmol amount of each light peptide in the sample. The peptide fmol amounts for each protein of interest were averaged for each sample, then normalized to the average fmol amount for actin within the same sample. The normalized protein/actin peak areas were then averaged for the four biological replicates of each age, giving an average protein/actin intensity measurement for each protein of interest. For the calibration curve samples, a linear regression of the heavy peptide peak area in each of the four calibration samples was performed and tested for linearity around the measurement range. Peptides that did not perform linearly (R2>0.98) were excluded from analysis.

### COS7 cell filopodia analysis

COS7 cells (ATCC CRL-1651; http://www.atcc.org/Products/All/CRL-1651) were trypsinized, plated on coverslips and maintained at 37 °C in DMEM supplemented with 10% fetal bovine serum. Cells were transfected using Lipofectamine transfection reagent (Invitrogen) according to manufacturer’s instructions and incubated for 24 h. Samples were then fixed for 20 min in 4% formaldehyde in PBS, permeabilized for 30 min in 0.5% Triton X-100 in PBS and counterstained or processed for immunofluorescence as described earlier. Relative pixel intensity of fluorescently tagged proteins along filopodia was determined using ImageJ (NIH) software. All measurements were performed using ImageJ. The mean grey value within a specified region of interest was used to represent the fluorescence intensity of mEmerald-ESPNL in each transfected cell.

### Homology analysis of the ARD domain of ESPN-1 and ESPNL

Protein sequences of the N-terminal ARD of mouse ESPN-1 (amino acids 1–331) and ESPNL (amino acids 1–334) were aligned using ClustalW2 (ref. 58). The estimated sequence homology for the ARD of both proteins was 54%. ESPN-1 and ESPNL protein secondary structure was predicted using PSIPRED^59^. Structural models of the ARD of mouse ESPN-1 and ESPNL proteins were generated using I-TASSER^60^ and the human ankyrin R protein as a template (PDB ID: 1n11). The models were aligned using the MatchMaker tool included in the Chimera software^61^. The root-mean-square deviation between the ESPN-1 and ESPNL structural model was of 1.76 Å for 330 residues.

### GST pull-down assays

Recombinant GST-ESPNL ARD protein was expressed in Rosetta cells and purified from bacterial lysates by using glutathione-agarose beads (Thermo Scientific). GFP-tagged MYO3A and MYO3B pre-THDI, THDI and post-THDI domain fusion proteins were expressed, respectively, in COS7 cells and lysates were prepared^13^. After 24 h incubation of COS7 cells following transfection with fusion constructs, lysates were prepared by brief sonication in ice-cold lysis buffer (CLB) (5 mM DTT, 50 mM Tris pH 7.4, 150 mM NaCl, 2 mM EDTA, 1% Triton X −100, 1 mM PMSF, 1 mM aprotinin and 1 mM leupeptin) and ultracentrifugation at 150,000*g* for 20 min. To test for interactions with MYO3A and MYO3B tail domains, the same amount of GST-ESPNL ARD or GST alone was bound to glutathione-agarose beads for 1 h at 4 °C, followed by incubation with COS7 cell lysate expressing GFP-tagged MYO3 A and MYO3B tail domain fusion proteins, respectively, in CLB for 2 h. The glutathione-agarose beads were then washed three times with 1 × PBS. The final pellets were then resuspended in SDS–polyacrylamide gel electrophoresis buffer.

Co-precipitates obtained from the final step of GST pull-down or COS7 lysate supernatants were separated on NuPAGE Bis-Tris 4–12% gels (Invitrogen) and transferred to nitrocellulose membrane for analysis by protein immunoblotting. Rabbit polyclonal anti-GFP antibodies (Invitrogen) were used to detect GFP-tagged MYO3A and MYO3B tail domain fusion proteins in the lysate and pull-down fractions; rabbit polyclonal anti-GST (Calbiochem) antibodies were used to detect GST-ESPNL ARD or GST alone in purified and GST pull-down fractions. All immunoblots were visualized using horseradish peroxidise-linked goat anti-rabbit secondary antibodies (Cell Signaling) and LumiGLO chemiluminescent substrate (Cell Signaling).

### Experiment repeats

Figure 1b–e: Confocal of *Espn-1*^−/−^ bundles. Experiment repeated at least six times. Figure 1f–j: SEM of *Espn-1*^−/−^ bundle. Experiment repeated two times, each three animals of each genotype. Figure 1k,l: TEM of *Espn-1*^−/−^ bundle. Experiment repeated two times, with three of each genotype. Figure 1n–p: Immunofluorescence of MYO3B in WT; immunofluorescence of MYO3B in *Espn-1*^−/−^; IF of PB538 on *Espn-1*^−/−^. Experiment repeated at least six times.

Figure 2a–h: Mass spectrometry. Repeats described in legend. Figure 2i–m: High-resolution stitched-panel montages of utricles labelled with antibodies against ESPN, ESPN-1, ESPNL, MYO3A and MYO3B. Experiment repeated at least three times each.

Figure 3a–f: Immunofluorescence of ESPN, ESPN-1 and ESPNL. Experiment repeated more than five times. Figure 3g,h: Super-resolution. Experiment repeated two times. Figure 3i: ESPNL-GFP. Experiment repeated four times.

Figure 4b–e: ESPN-1 and ESPNL COS7 transfections. Experiment repeated more than five times. Figure 4g: Antibody testing. Experiment repeated once by immunostaining and once by protein immunoblot. Figure 4j–k: SEM of *Espnl* knockout. Experiment repeated two times.

Figure 5d,e: ESPNL-ARD+MYO3A and ESPNL-ARD+MYO3B. Experiment repeated more than three times.

Figure 6a,d,e: Immunofluorescence of MYO3A and MYO3B. Experiment repeated more than six times. Figure 6b,c: Confocal and SIM of MYO3A. Experiment repeated two times. Figure 6h,j: PCR with reverse transcription (RT–PCR). Experiment repeated two times. Figure 6i,k: ABR. Repeat information in legend. Figure 6l–n: confocal of bundle morphology in *Myo3a* and *Myo3b* null mice. Experiment repeated more than six times, with total numbers of bundles examined listed in the legend.

Figure 7. COS7 transfections were all repeated more than six times.

## Additional information

**How to cite this article:** Ebrahim, S. *et al.* Stereocilia staircase spacing is influenced by myosin III motors and their cargos espin-1 and espin-like. *Nat. Commun.* 7:10833 doi: 10.1038/ncomms10833 (2016).

## Supplementary Material

Supplementary Information Supplementary Figures 1-13 and Supplementary Table 1
