# Corrigendum: Stereocilia-staircase spacing is influenced by myosin III motors and their cargos espin-1 and espin-like

**DOI:** 10.1038/ncomms16133

**Published:** 2017-08-14

**Authors:** Seham Ebrahim, Matthew R. Avenarius, M’hamed Grati, Jocelyn F. Krey, Alanna M. Windsor, Aurea D. Sousa, Angela Ballesteros, Runjia Cui, Bryan A. Millis, Felipe T. Salles, Michelle A. Baird, Michael W. Davidson, Sherri M. Jones, Dongseok Choi, Lijin Dong, Manmeet H. Raval, Christopher M. Yengo, Peter G. Barr-Gillespie, Bechara Kachar

Nature Communications 7 Article number: 10833 ; DOI: 10.1038/ncomms10833 (2016). Published 03 01 2016; Updated 08 14 2017

Technical support for this Article was not fully acknowledged. The Acknowledgements should have included the following: We thank Drs Ronald Petralia and Ya-Xian Wang, members of the Advanced Imaging Core, National Institute of Deafness and other Communication Disorders (NIDCD), for the images in Fig. 1k, l.

