# Supplementary material for: Stereocilia-staircase spacing is influenced by myosin III motors and their cargos espin-1 and espin-like
Source: Nat Commun. 2016 Mar 1;7:10833. doi: 10.1038/ncomms10833 (PMC4773517; doi:10.1038/ncomms10833)
Supplement: Supplementary Information — Supplementary Figures 1-13 and Supplementary Table 1 [file ncomms10833-s1.pdf]

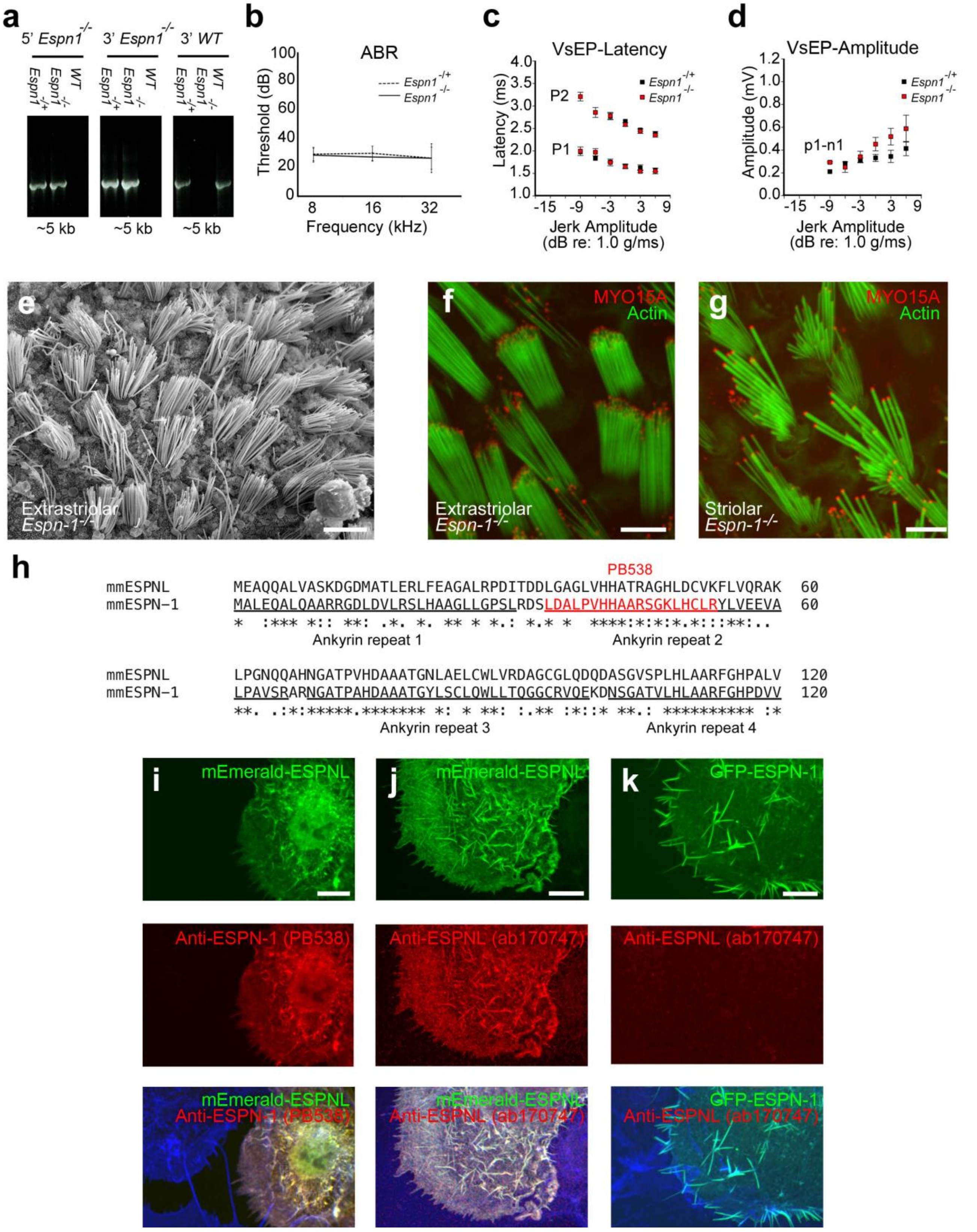

Supplementary Figure 1. *Espn-1* knockout characterization. (**a**) The predicted recombinant *Espn-1*$^{-/-}$ allele was detected by PCR of the left (5') homologous recombination arm (left) and of the right (3') homologous recombination arm (middle). The wild-type non-recombinant allele was detected by PCR using a forward primer on E1 and a reverse primer downstream of the right arm homologous recombination genomic sequence (right). (**b**) Mean ABR measurements (± SEM) from *Espn-1*$^{-/-}$ and wild-type mice at six months (each n=9).

(**c-d**) Mean vestibular evoked potential (VsEP) measurements (± standard error) for *Espn-1*$^{-/-}$ (n=6) and heterozygous littermates (n=2). No significant differences were observed between genotypes for response peak P1 and P2 latencies (c) or P1–N1 amplitudes (d). VsEP thresholds were -6.5 ± 5.9 dB for *Espn-1*$^{-/-}$ and -7.5 ± 4.2 dB for heterozygous littermates. (**e**) SEM image of extrastriolar hair bundles of a six-month-old *Espn-1*$^{-/-}$ mouse. (**f-g**) Confocal fluorescence micrographs of stereocilia from vestibular hair cells from the extrastriolar (f) and striolar (g) regions of the utricle from 1-year old *Espn-1*$^{-/-}$ mouse. Phalloidin conjugated to AlexaFluor-488 was used to label actin (green); the anti-MYO15A antibody (red) shows that MYO15A label persists at stereocilia-tips in both affected and unaffected bundles. (**h**) Alignment of the first four ankyrin repeats of ESPN-1 and ESPNL. The PB538 antibody epitope is highlighted in red. (**i-j**) COS7 cells transfected with mEmerald-ESPNL (green), and labeled with an anti-ESPN-1 (i) or anti-ESPNL (j) antibody to test for selectivity and specificity of antibodies used. Both PB538 (red) and anti-ESPNL (red) antibody label mEmerald-ESPNL. (**k**) The anti-ESPNL (red) antibody did not label GFP-ESPN-1 (green) expressed in COS7 cells. Scale bars: 5 µm.

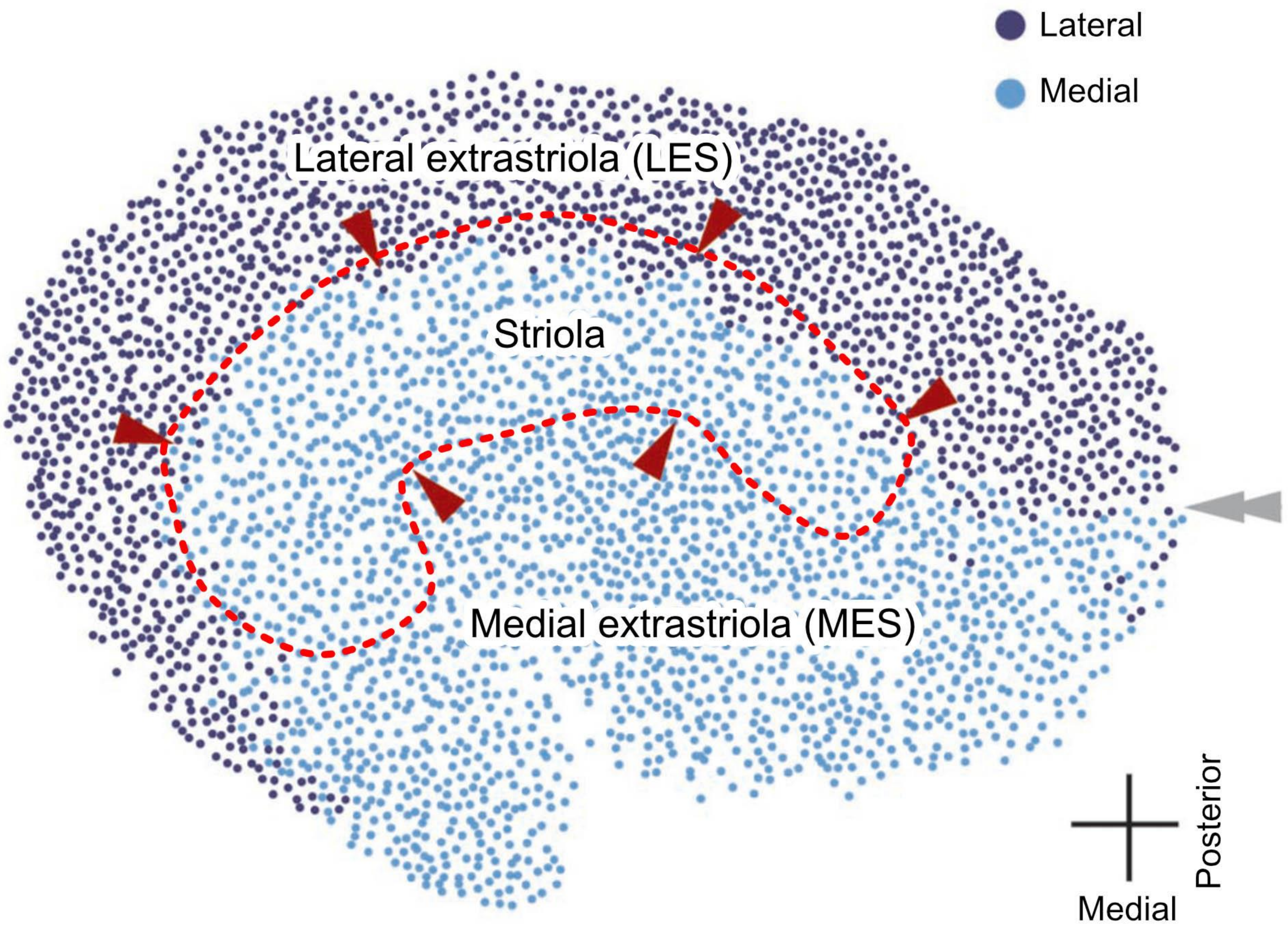

Supplementary Figure 2. Morphology of the mouse utricle. Figure depicts the hair cells of a single utricle; each dot corresponds to one hair cell. Purple depicts lateral hair cells and light blue medial hair cells; at the boundary between the two zones, the orientation of the hair bundles reverses. The striola is a central region of the utricle with reduced hair cell density and calretinin-positive vestibular nerve calyces surrounding type I hairs; in this figure, the striola is demarked both by dark red arrowheads and by the red dashed line. Hair cells outside the striola are referred to as extrastriolar, and there are medial and lateral populations of these cells. Unlike in other vertebrates, rodent utricles have type I and type II hair cells distributed both in striolar and extrastriolar regions. Figure modified from Figure 2B of Li et al. (2008) *J. Neurophysiol.* **99**, 718–733, with permission.

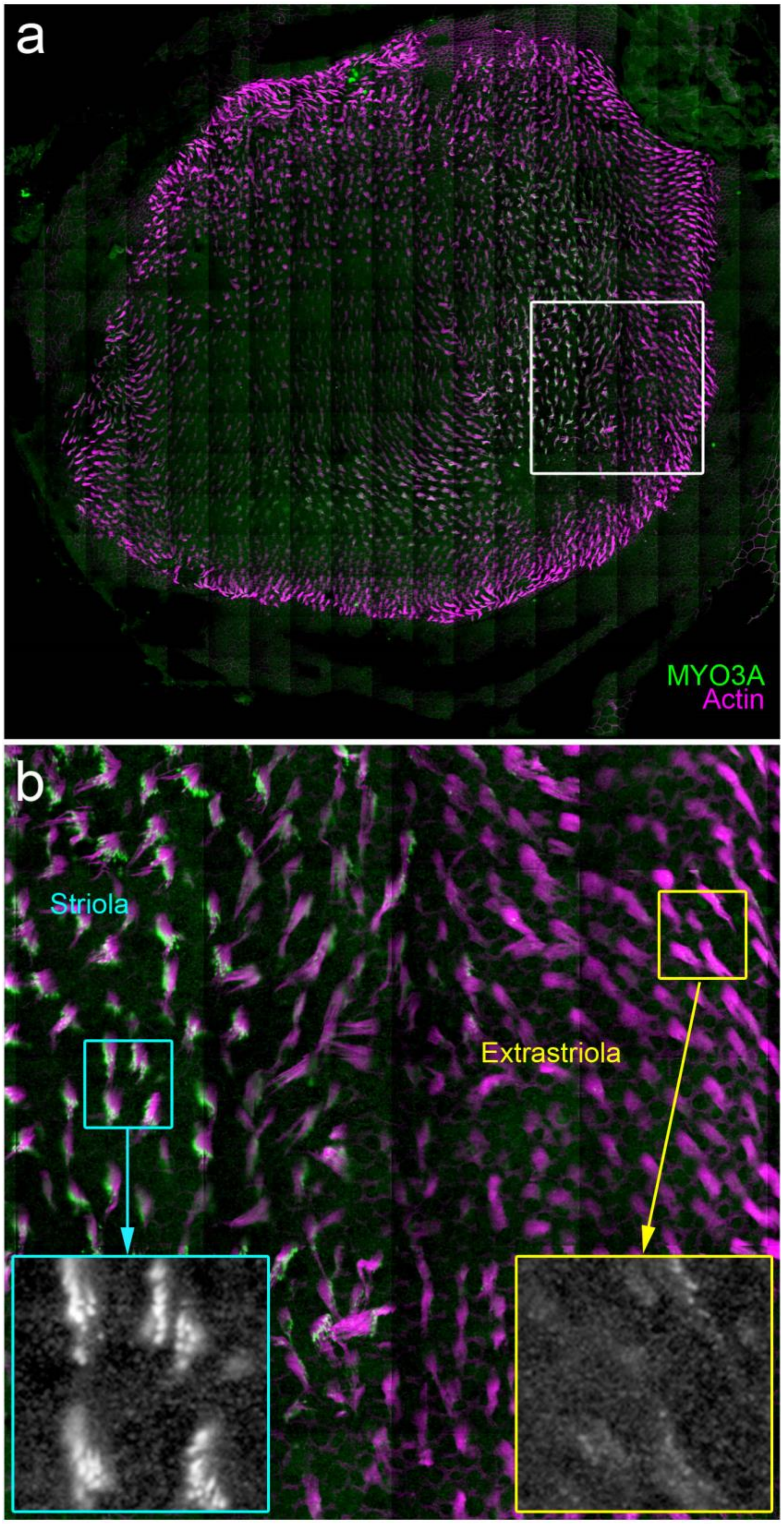

Supplementary Figure 3. High-resolution view of MYO3A in the striolar region of the utricle. (**a**) Survey of utricle at low resolution. (**b**) Magnification of region indicated by white box in panel (a). Striolar region in small cyan box is magnified 3x in large cyan box; extrastriolar region in small yellow box is magnified 3x in large yellow box. While MYO3A is substantially higher in striolar hair bundles, lower levels of MYO3A can be detected at stereocilia tips in extrastriolar hair cells as well (panel b, lower right yellow-boxed panel). Scale: white box in (a) is 216 µm across, as is full width of (b); cyan and yellow boxes in (b) are 24 µm across.

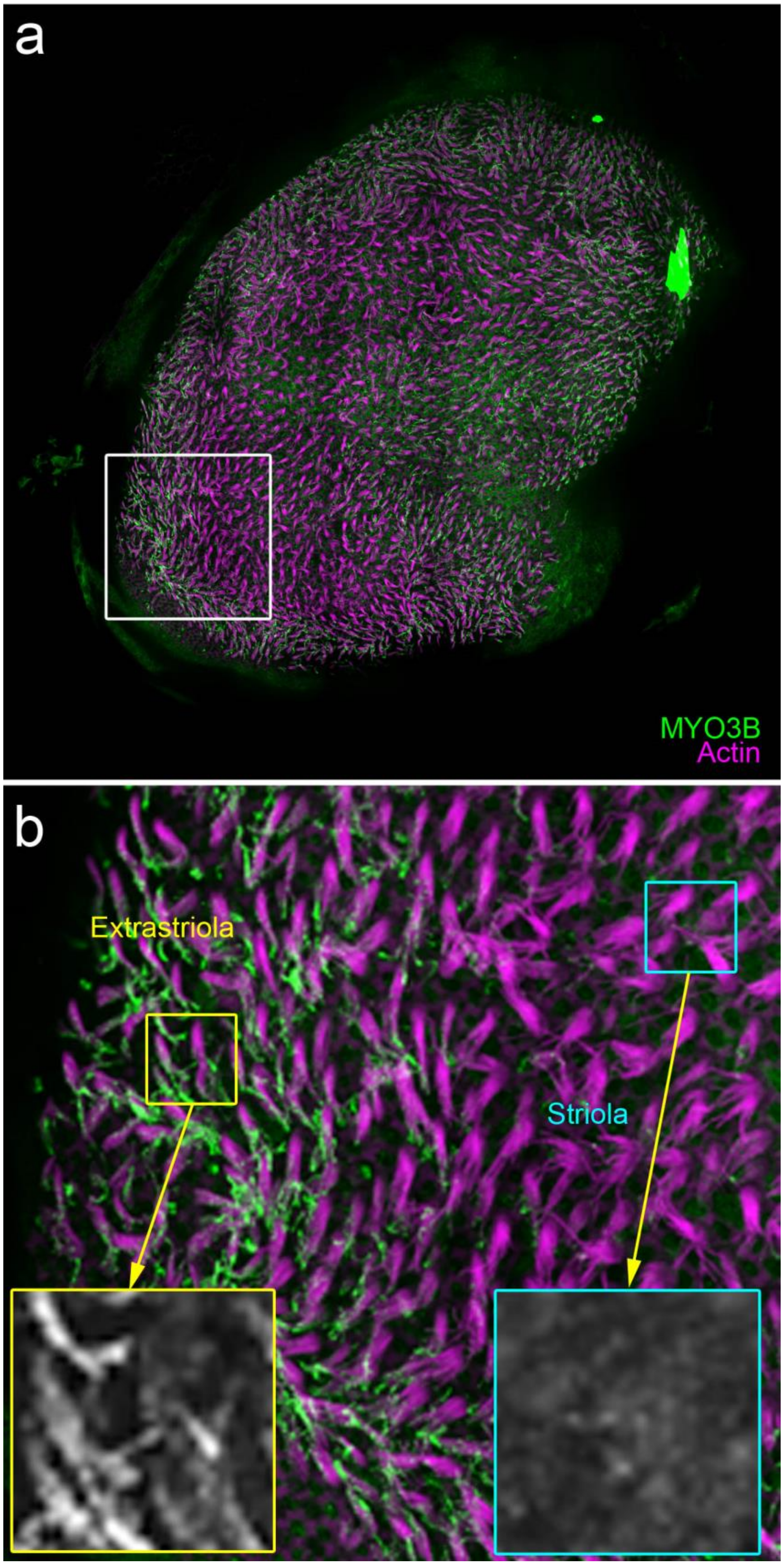

Supplementary Figure 4. High-resolution view of MYO3B in the extrastriolar region of the utricle. Same figure layout as Supplementary Figure 3. While MYO3B is substantially higher in extrastriolar hair bundles, very low levels of MYO3B can be detected at stereocilia tips in extrastriolar hair cells as well (panel b, lower right cyan-boxed panel). Scale: white box in (a) is 155 µm across, as is full width of (b); cyan and yellow boxes in (b) are 18 µm across.

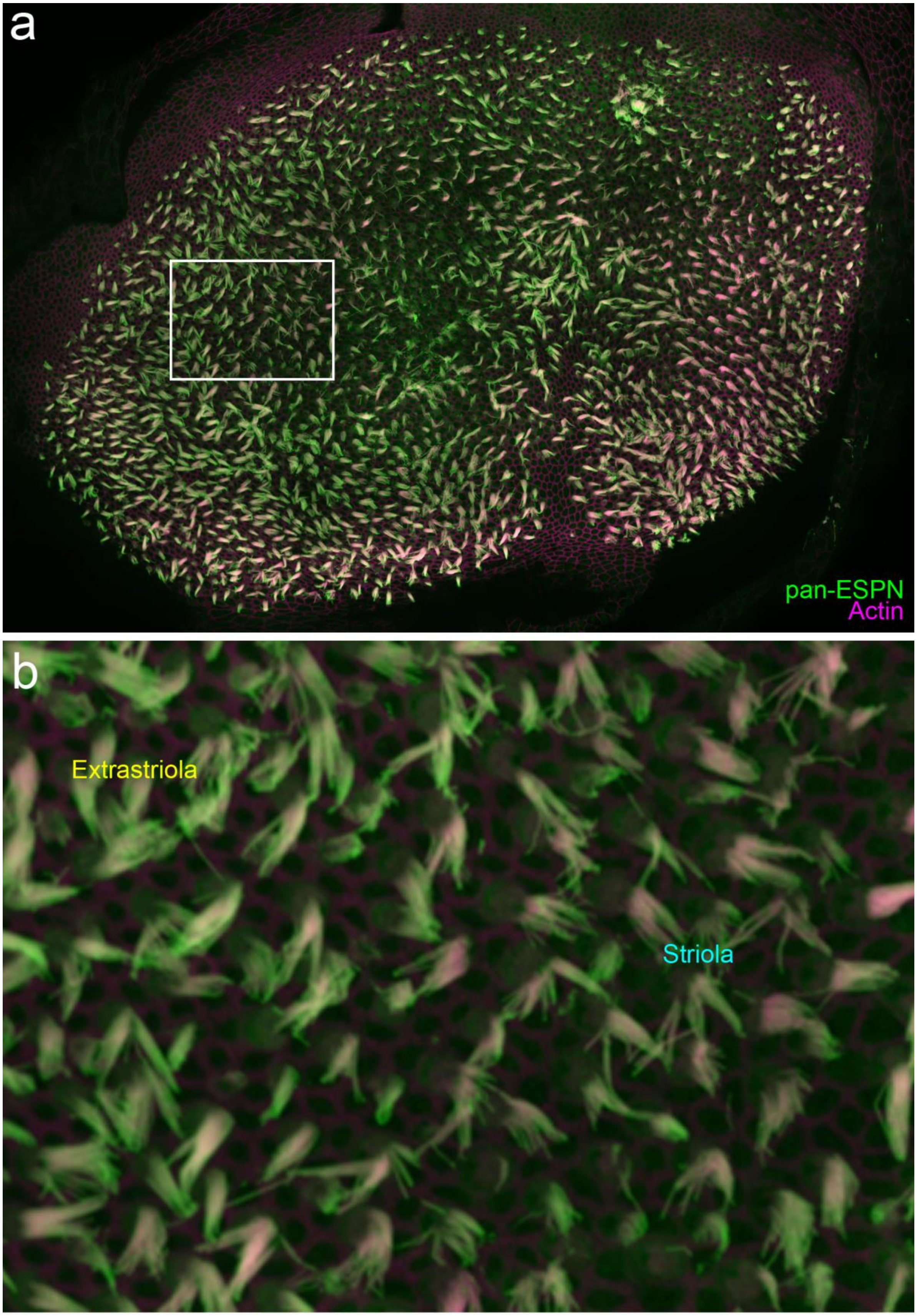

Supplementary Figure 5. High-resolution view of ESPN in the utricle. Same figure layout as Supplementary Figure 3. Labeling with pan-ESPN antibody appears similar in striolar and extrastriolar regions. Scale: white box in (a) is 121 µm across, as is (b).

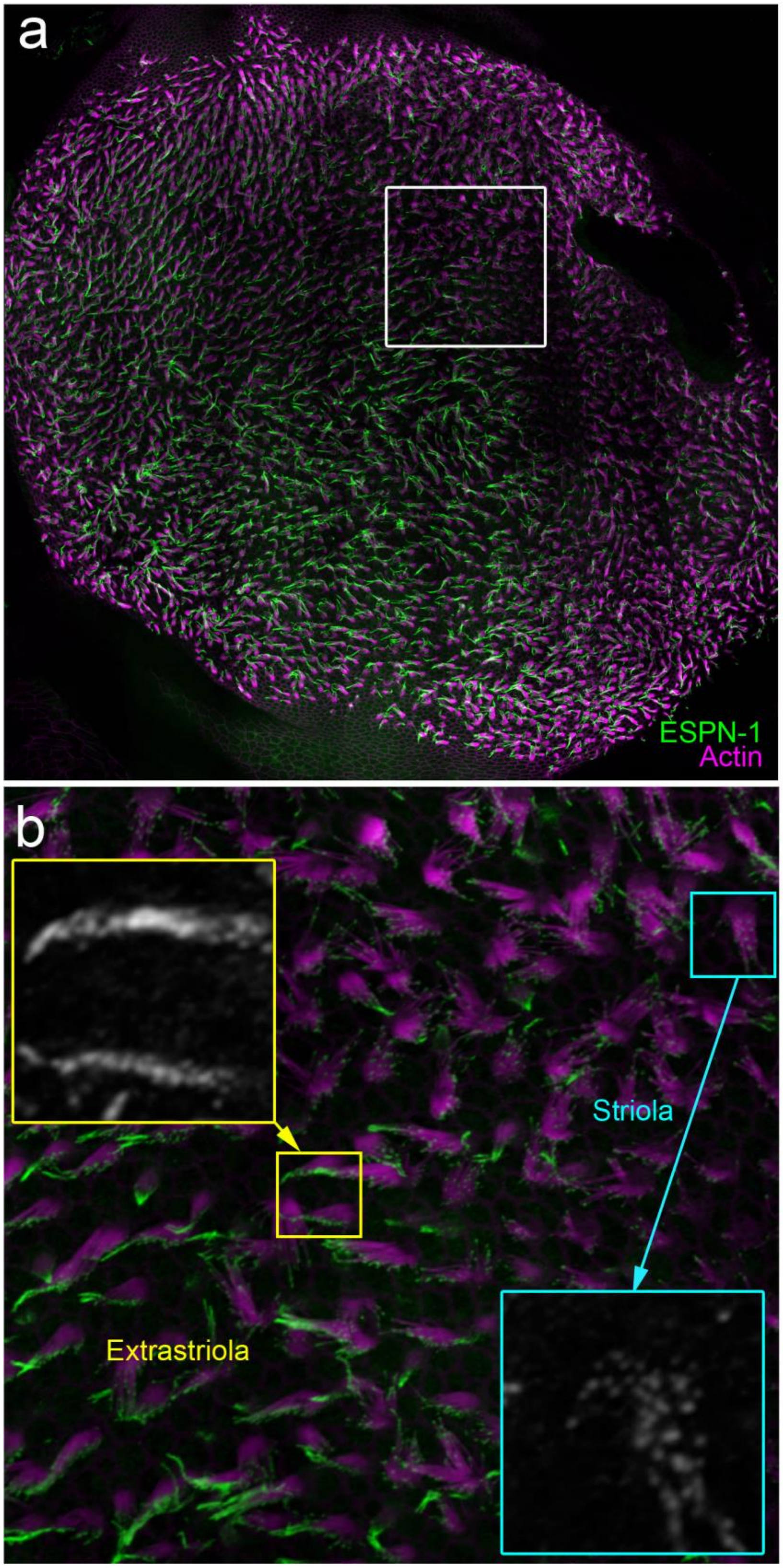

Supplementary Figure 6. High-resolution view of ESPN-1 in the extrastriolar region of the utricle. Same figure layout as Supplementary Figure 3. While ESPN-1 is substantially higher in extrastriolar hair bundles, lower levels of ESPN-1 can be detected at stereocilia tips in in striolar hair cells as well (panel b, lower right yellow-boxed panel). Scale: white box in (a) is 143 µm across, as is full width of (b); cyan and yellow boxes in (b) are 16 µm across.

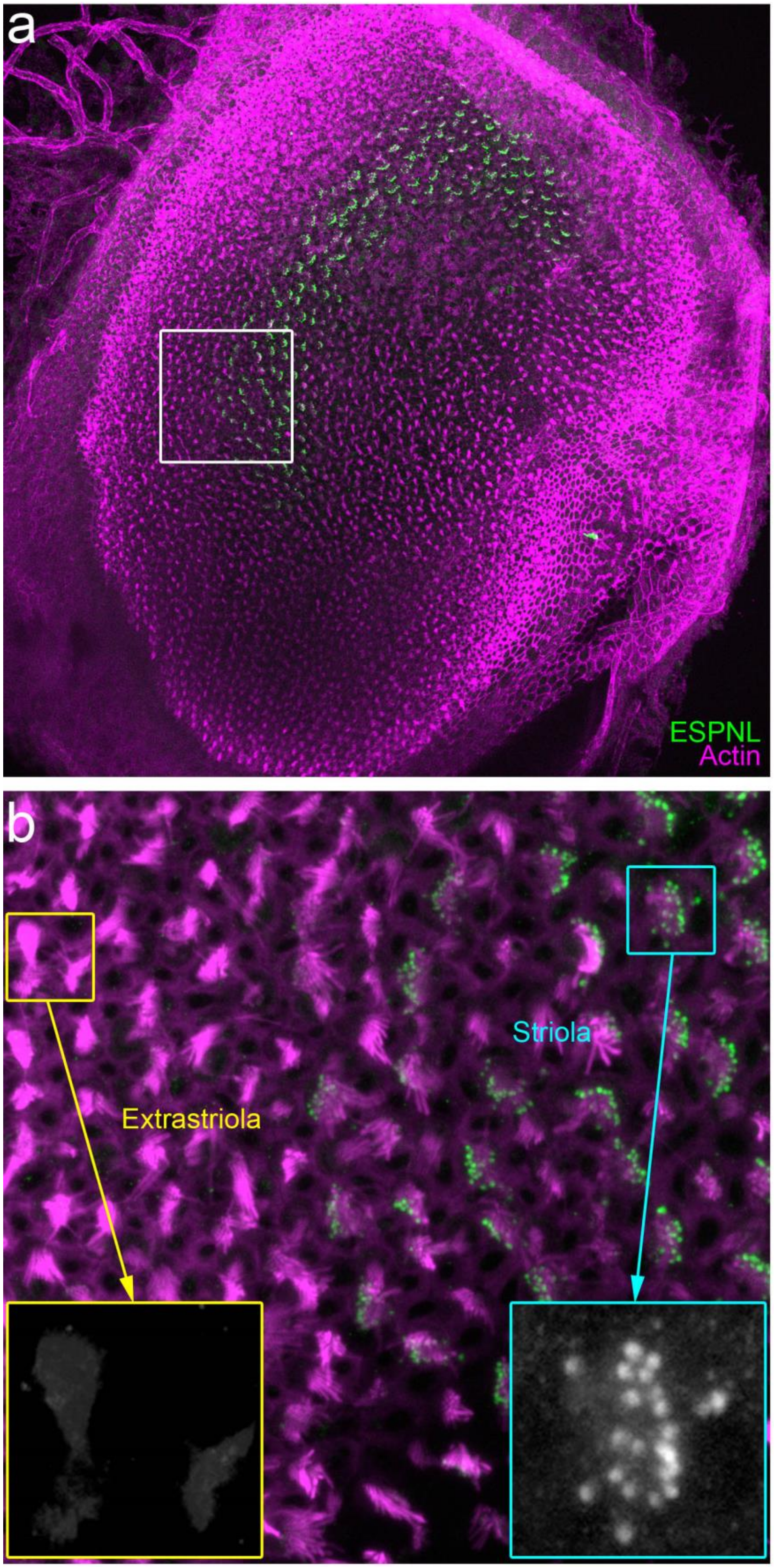

Supplementary Figure 7. High-resolution view of the concentration of ESPNL in the striolar region of the utricle. Same figure layout as Supplementary Figure 3. ESPNL appears to be highly specific for the striola. Scale: white box in (a) is 98 µm across, as is full width of (b); cyan and yellow boxes in (b) are 12 µm across.

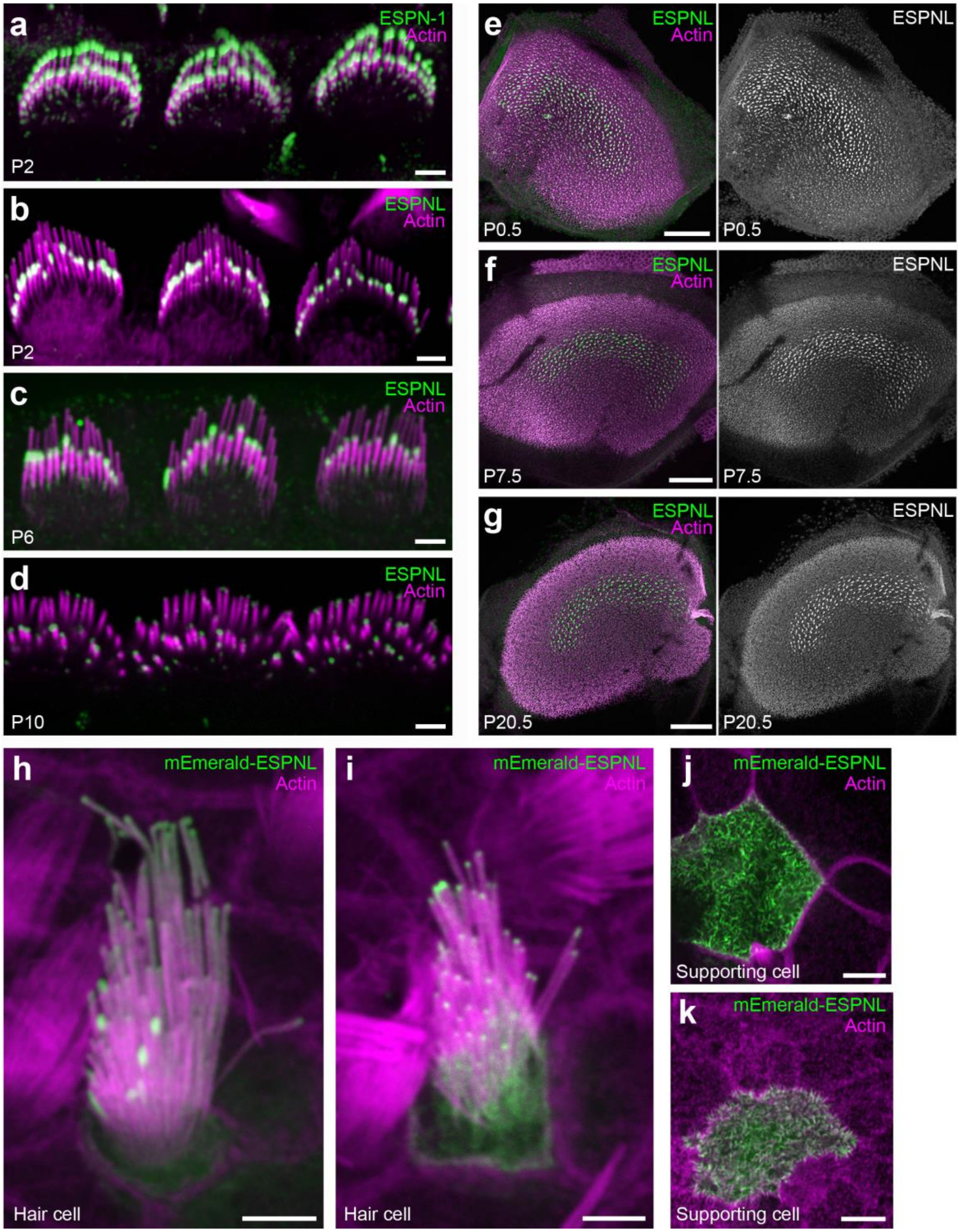

Supplementary Figure 8. Development of ESPNL immunoreactivity in cochlear and vestibular hair cells. (**a**) ESPN-1 labeling (green) in P2 cochlear inner hair cells. (**b-d**) Developmental progression of ab170747 ESPNL labeling (green) in P2-P10 cochlear inner hair cells. (**e-g**) Developmental progression of BG35961 ESPNL labeling (left, green; right, gray) in P0.5-P20.5 utricles. (**h-i**) Confocal fluorescence images of stereocilia from vestibular hair cells of rats (P2), expressing ESPNL-mEmerald (green) after gene gun-mediated biolistic transfection, shows ESPNL localization to stereocilia and concentration at stereocilia-tips. (**j-k**) Supporting cells from rat organ of Corti of rat (P2) expressing ESPNL-mEmerald (green) shows a concentration of ESPNL at actin-based microvilli. Phalloidin conjugated to AlexaFluor-568 was used to label actin (magenta). Scale bars: A-D, H-K, 2 µm; E-G, 100 µm.

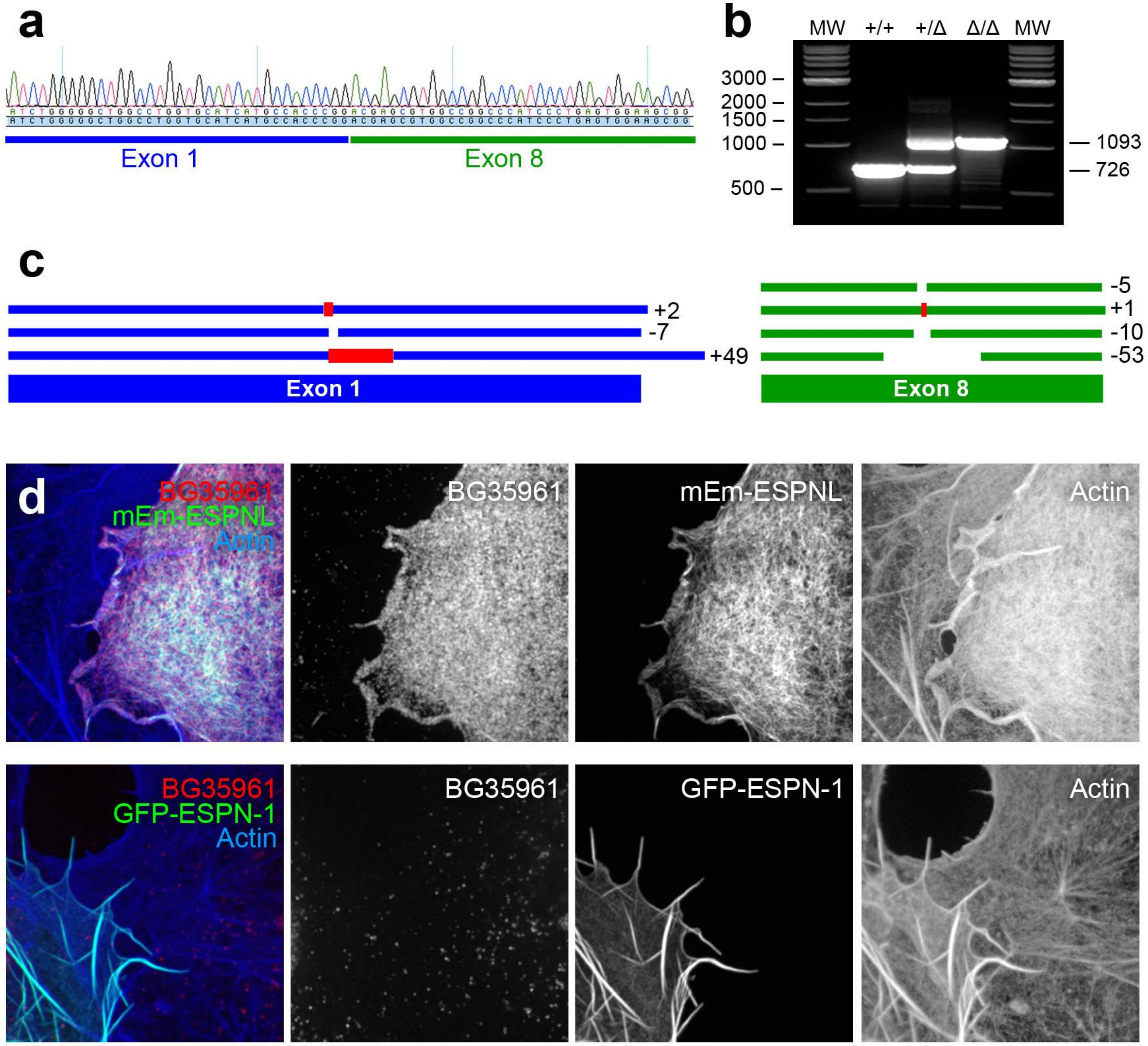

Supplementary Figure 9. Characterization of *Espnl* mutations and phenotype, and validation of BG35961 antibody. (**a**) Sequencing chromatogram for *Espnl*$^{\Delta}$ allele showing breakpoint between exons 1 and 8. (**b**) PCR genotyping of *Espnl*$^{\Delta}$ allele. Expected sizes for *Espnl*$^{\Delta}$ and wild-type products are indicated on right. (**c**) Diagrammatic representation of several characterized CRISPR-mediated indel alleles of Espnl. Numbers beside each smaller bar (allele) are the net change in nucleotides in the indicated exon. Red bars are insertions. Only alleles that were distinguished during sequencing runs were illustrated; other genotyping assays provided evidence for mosaic expression of multiple alleles. (**d**) Validation of BG35961 anti-ESPNL antibody. Cells were transfected with either mEmerald-ESPNL or GFP-ESPN-1, and then were stained with the BG35961 antibody and phalloidin for actin. Note that BG35961 stains the transfected cell in the top row, but not the adjacent untransfected cell. BG35961 stains neither transfected nor untransfected cells in the GFP-ESPN-1 experiment. Each panel is 24.5 µm wide and tall.

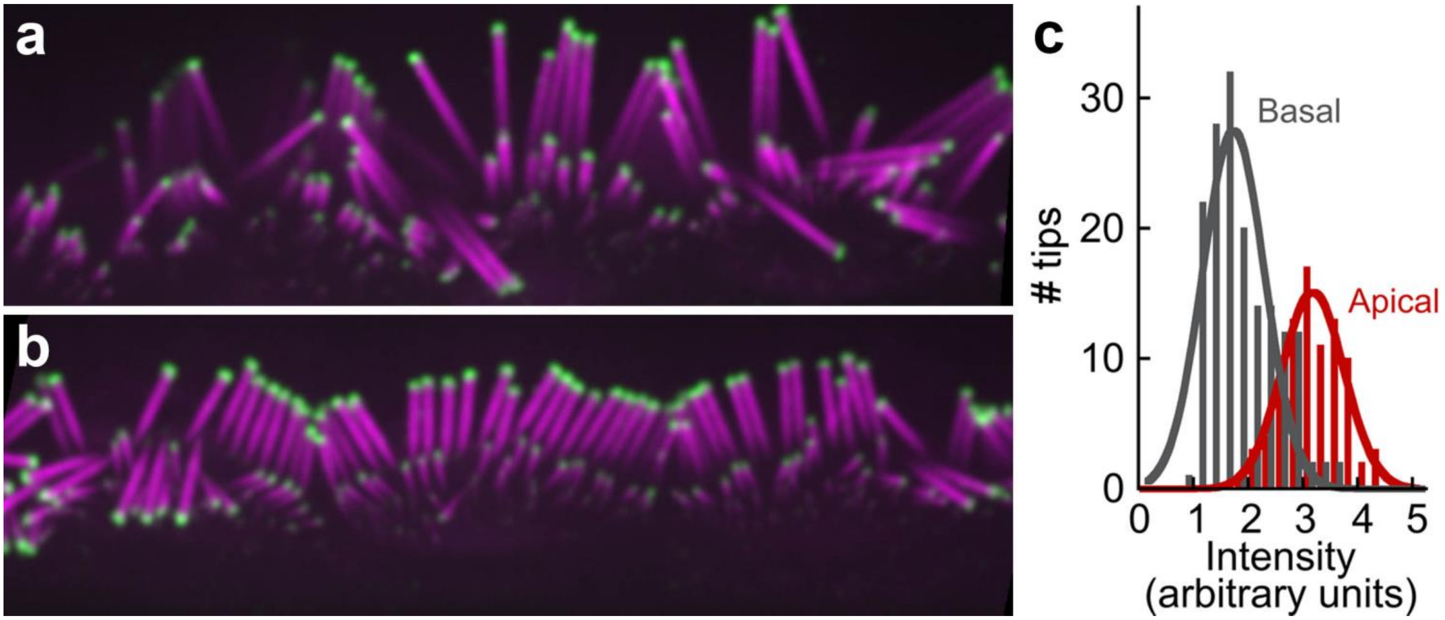

Supplementary Figure 10. ESPN-1 expression in stereocilia of apical and basal stereocilia from P9 cochleas. (**a**) Apical. (**b**) Basal. A and B are 30 µm wide and 9 µm tall. (**c**) Distribution of signal intensity for 85 (apical) and 161 (basal) stereocilia tips. The apical mean was 2.58 ± 0.05 arbitrary units (n=85); the basal mean was 1.59 ± 0.04 arbitrary units (n=161); applying a Student's t-test, $p = 10^{-44}$.

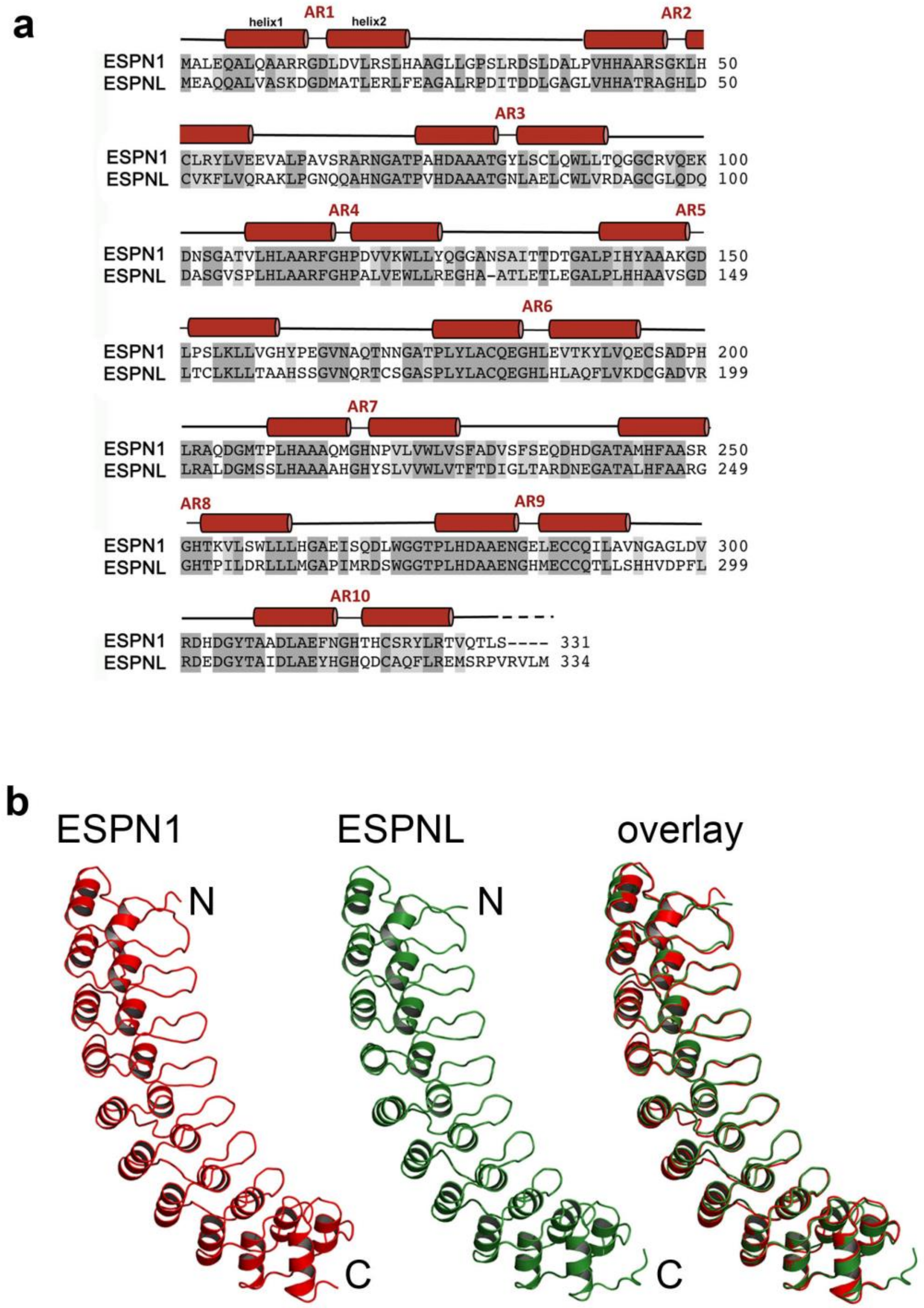

Supplementary Figure 11. Structural similarities of ESPN-1 and ESPNL ankyrin-repeat domains. (**a**) Sequence alignment of mouse ESPN1 and ESPNL N-terminal ankyrin-repeat domain (ARD) colored by sequence identity. Conserved and similar amino acids are highlighted in dark and light gray respectively. Secondary structure is shown schematically above the amino acid sequences, with red cylinders for α-helix and black lines for loops. The ten ARs consisting of two α-helices (helix1 and helix2) separated by loops are numbered. (**b**) Structural alignment of the ARD of ESPN1 (red) and ESPNL (green), and overlay (right). Homology analysis is described in Supplemental Experimental Procedures.

AaBb x AaBb (24 progeny)

	Parent 2: AB	Ab	aB	ab
**Parent 1: AB**	AA BB	AA Bb	Aa BB	Aa Bb
**Ab**	AA Bb	AA bb	Aa Bb	**Aa bb**
**aB**	Aa BB	Aa Bb	aa BB	**aa Bb**
**ab**	Aa Bb	**Aa bb**	**aa Bb**	**aa bb**

AaBb x aaBb (39 progeny)

	Parent 2: aB	ab	aB	ab
**Parent 1: AB**	Aa BB	Aa Bb	Aa BB	Aa Bb
**Ab**	Aa Bb	**Aa bb**	Aa Bb	**Aa bb**
**aB**	aa BB	**aa Bb**	aa BB	**aa Bb**
**ab**	**aa Bb**	**aa bb**	**aa Bb**	**aa bb**

aaBb x aaBb (25 progeny)

	Parent 2: aB	ab	aB	ab
**Parent 1: AB**	aa BB	**aa Bb**	aa BB	**aa Bb**
**Ab**	**aa Bb**	**aa bb**	**aa Bb**	**aa bb**
**aB**	aa BB	**aa Bb**	aa BB	**aa Bb**
**ab**	**aa Bb**	**aa bb**	**aa Bb**	**aa bb**

Overall (88 progeny)

**Geno-type**	**Expected**	**Obtained**
AA BB	1.5	1
AA Bb	3.0	7
Aa BB	7.9	5
Aa Bb	15.8	26
aaBB	12.6	14
AA bb	1.5	1
**aa Bb**	25.3	34
**Aa bb**	7.9	**0**
**aa bb**	12.6	**0**

A = *Myo3a*$^+$, a = *Myo3a*$^-$; B = *Myo3b*$^+$, b = *Myo3b*$^-$

Supplementary Figure 12. Punnett squares indicating expected genotypes arising from three crosses designed to generate myosin-III double-knockout mice. Right, expected numbers of each genotype and observed numbers.

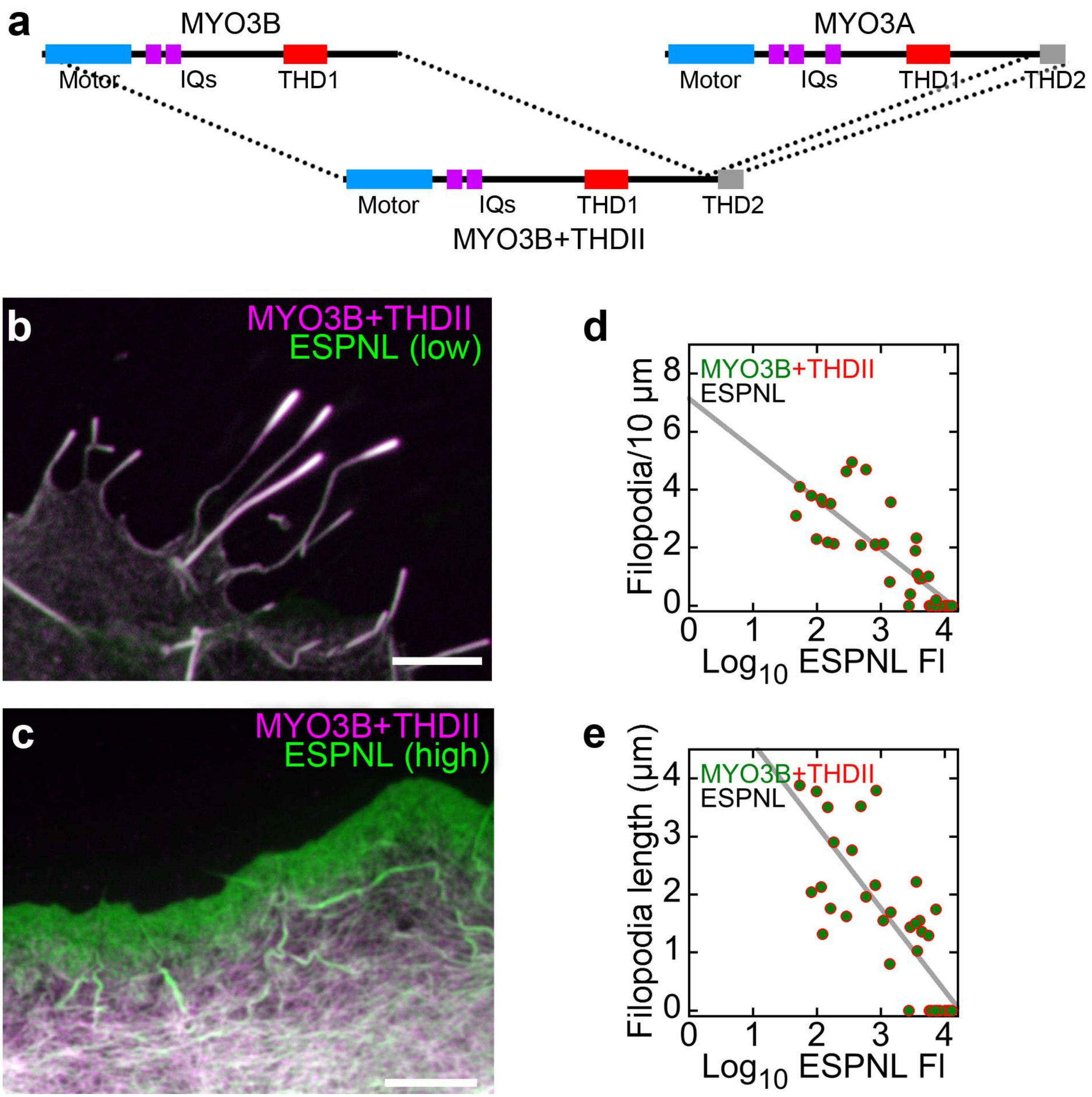

Supplementary Figure 13. MYO3B-ESPNL mimics MYO3A-ESPNL when MYO3A THDII is added to MYO3B. (**a**) Diagram of MYO3B hybrid protein containing the THDII of MYO3A (mCherry-MYO3B+THDII). (**b**) mCherry-MYO3B+THDII mimics the behavior of MYO3A when co-expressed with ESPNL, co-translocating to filopodia tips in cells expressing relatively low levels of mEmerald-ESPNL. (**c**) The hybrid protein is unable to initiate filopodia in cells expressing high levels of mEmerald-ESPN. (**d-e**) Quantitation of relationship between ESPNL fluorescence level (ESPNL FI) and filopodia density (d) or length (e). There was an inverse relationship of filopodial number and length with ESPNL fluorescence for MYO3B+THDII (r=0.82 and 0.79). Scale bars: 2 µm.

Supplementary Table 1. Two-way ANOVA analysis of filopodia length and number. The data from either MYO3A or MYO3B alone are the reference group. Then, two main effects estimates for +EPSN-1 and +ESPNL effects are added to the reference. Finally, the fourth parameter estimates the interaction of both (+ESPN-1 +ESPNL).

MYO3A group, Figure 7o:

	Estimate	Std. Error	t value	p-value
MYO3A+ (reference)	3.05	0.27	11.16	<0.001
+ESPN-1	0.31	0.38	0.80	0.427
+EPSNL	-1.96	0.35	-5.61	<0.001
+Interaction	2.53	0.54	4.70	<0.001

MYO3B group, Figure 7o:

	Estimate	Std. Error	t value	p-value
MYO3B+ (reference)	0.67	0.22	3.04	0.003
+ESPN-1	1.92	0.28	6.96	0.000
+EPSNL	2.54	0.29	8.66	0.000
+Interaction	-1.35	0.42	-3.24	0.002

MYO3A group, Figure 7p:

	Estimate	Std. Error	t value	p-value
MYO3A+ (reference)	1.79	0.30	5.98	<0.001
+ESPN-1	4.95	0.42	11.81	<0.001
+EPSNL	-0.35	0.38	-0.90	0.369
+Interaction	-3.13	0.59	-5.30	0.000

MYO3B group, Figure 7p:

	Estimate	Std. Error	t value	p-value
MYO3B+ (reference)	1.52	0.69	2.22	0.029
+ESPN-1	3.28	0.87	3.79	<0.001
+EPSNL	1.43	0.91	1.57	0.121
+Interaction	3.77	1.30	2.91	<0.001
